# OXA1L deficiency causes mitochondrial myopathy via reactive oxygen species regulated nuclear factor kappa B signalling pathway

**DOI:** 10.1002/ctm2.70385

**Published:** 2025-06-23

**Authors:** Yongkun Zhan, Qian Wang, Ya Wang, Yanjie Fan, Dan Yan, Xianlong Lin, Yaoting Chen, Tingting Hu, Nan Li, Weiqian Dai, Hezhi Fang, Yongguo Yu

**Affiliations:** ^1^ Department of Clinical Genetics Center Shanghai Institute for Pediatric Research Xinhua Hospital affiliated to Shanghai Jiao Tong University School of Medicine Shanghai China; ^2^ Department of Clinical Laboratory Center The Second Affiliated Hospital & Yuying Children's Hospital of Wenzhou Medical University Wenzhou Zhejiang China; ^3^ Key Laboratory of Laboratory Medicine, School of Laboratory Medicine and Life Sciences, Wenzhou Medical University Wenzhou Zhejiang China; ^4^ Department of Clinical Laboratory State Key Laboratory of Molecular Oncology, National Cancer Center/National Clinical Research Center for Cancer/Cancer Hospital, Chinese Academy of Medical Sciences and Peking Union Medical College Beijing China; ^5^ Department of Pediatric Endocrinology and Genetics Xinhua Hospital Affiliated to Shanghai Jiao Tong University School of Medicine Shanghai China

**Keywords:** mitochondrial myopathy, NF‐κB signalling pathway, OXA1L, oxidative phosphorylation, reactive oxygen species

## Abstract

**Background:**

OXA1L is crucial for mitochondrial protein insertion and assembly into the inner mitochondrial membrane, and its variants have been recently linked to mitochondrial encephalopathy. However, the definitive pathogenic link between *OXA1L* variants and mitochondrial diseases as well as the underlying pathogenesis remains elusive.

**Methods:**

In this study, we identified bi‐allelic variants of c.620G>T, p.(Cys207Phe) and c.1163_1164del, p.(Val388Alafs*15) in *OXA1L* gene in a mitochondrial myopathy patient using whole exome sequencing. To unravel the genotype–phenotype relationship and underlying pathogenic mechanism between *OXA1L* variants and mitochondrial diseases, patient‐specific human‐induced pluripotent stem cells (hiPSC) were reprogrammed and differentiated into myotubes, while *OXA1L* knockout human immortalised skeletal muscle cells (IHSMC) and a conditional skeletal muscle knockout mouse model was generated using clustered regularly interspaced short palindromic repeats/Cas9 genomic editing technology.

**Results:**

Both patient‐specific hiPSC differentiated myotubes and *OXA1L* knockout IHSMC showed combined mitochondrial respiratory chain defects and oxidative phosphorylation (OXPHOS) impairments. Notably, in *OXA1L*‐knockout IHSMC, transfection of wild‐type human OXA1L but not truncated mutant form rescued the respiratory chain defects. Moreover, skeletal muscle conditional *Oxa1l* knockout mice exhibited OXPHOS deficiencies and skeletal muscle morphofunctional abnormalities, recapitulating the phenotypes of mitochondrial myopathy. Further functional investigations revealed that impaired OXPHOS resulting of OXA1L deficiency led to elevated reactive oxygen species production, which possibly activated the nuclear factor kappa B signalling pathway, triggering cell apoptosis.

**Conclusions:**

Together, our findings reinforce the genotype–phenotype association between *OXA1L* variations and mitochondrial diseases and further delineate the potential molecular mechanisms of how OXA1L deficiency causes skeletal muscle deficits in mitochondrial myopathy.

**Keypoints:**

1. *OXA1L* gene bi‐allelic variants cause mitochondrial myopathy.
2. OXA1L deficiency results in combined mitochondrial respiratory chain defects and OXPHOS impairments.
3. OXA1L deficiency leads to elevated ROS production, which may activate the NF‐κB signalling pathway, disturbing myogenic gene expression and triggering cell apoptosis.

## Graphical abstract

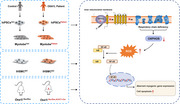

## 1 INTRODUCTION

Mitochondria, indispensable cytoplasmic organelles, are crucial for energy production. They serve as the cellular powerhouses, mediating the majority of ATP synthesis via oxidative phosphorylation (OXPHOS). The OXPHOS system relies on the electron transport chain, which encompasses five mitochondrial respiratory chain (MRC) complexes within the inner mitochondrial membrane.^1^ Moreover, mitochondria participate in numerous other vital processes, including reactive oxygen species (ROS) generation, calcium homeostasis maintenance and apoptotic cascades modulation,^2^, ^3^ all of which have gained recognition for their involvement in pathological processes.

Mitochondrial diseases, affecting approximately one in 5000 live births, are a heterogeneous spectrum of inherited metabolic disorders stemming from pathogenic variants in mitochondrial DNA (mtDNA)/nuclear DNA (nDNA) that impair OXPHOS.^4^, ^5^ The clinical features and severity of these diseases can vary widely, ranging from mild symptoms to severe multisystemic manifestations, typically involving high‐energy‐demand tissues, such as the central nervous system, skeletal muscles and heart.^6^, ^7^, ^8^ Despite advancements in diagnostic methods, early clinical recognition of patients with mitochondrial disorders remains a challenge.^9^, ^10^

Substantial advancements has been achieved in elucidating the genetic basis of mitochondrial diseases in recent decades.^10^, ^11^ Initially, mtDNA variations were primarily investigated, as they were considered the main culprits. However, with the advancement of next‐generation sequencing technologies, especially whole exome sequencing (WES), attention has shifted towards nDNA‐encoded components governing MRC complex assembly, mitochondrial translation systems and mtDNA replication and repair.^10^, ^11^, ^12^ The discovery of new disease‐causing genes has considerably expanded our understanding of mitochondrial diseases and their underlying molecular mechanisms. Despite the rapid expansion of the list of gene mutations identified in patients with primary mitochondrial disorders, given the complexity of mitochondrial architecture and genetic regulation, coupled with the genotype–phenotype heterogeneity of the mitochondrial diseases, there are still numerous candidate pathogenic genes to be explored.

The oxidase, cytochrome *c*, assembly 1‐like gene (*OXA1L*, MIM*601066), located on chromosome 14q11.2, encodes a mitochondrial inner membrane protein belonging to the YidC/Alb3/Oxa1 superfamily, which mediates the integration of nDNA and mtDNA‐encoded proteins into the mitochondrial inner membrane and facilitates folding and assembly of membrane proteins.^13^, ^14^, ^15^ Previous studies based on yeast have revealed that Oxa1p is essential for the synthesis and intramembrane trafficking of multiple MRC subunits.^16^, ^17^, ^18^ Genetic deletion of Oxa1 in yeast revealed severe reduction in complexes I (CI), III (CIII), IV (CIV) and V (CV).^19^ Similarly, interfering with OXA1L expression in human models has been shown to decrease the levels of CI, CIII, CIV and CV.^20^, ^21^ Though *OXA1L* has traditionally been included in diagnostic panels for combined MRC defects, there is presently only one publication linking *OXA1L* gene variant to mitochondrial encephalomyopathy.^21^ The association between *OXA1L* gene variation and mitochondrial disease as well as the underlying pathophysiological mechanisms remains unclear, necessitating further exploration through additional clinical cases and functional experiments.

In this study, we reported the second family with mitochondrial myopathy harbouring bi‐allelic variants in the *OXA1L* gene, broadening mutation spectrum and providing new evidence for the association between *OXA1L* variants and mitochondrial diseases. Functional investigations revealed combined MRC defects and impairment of multiple OXPHOS functions in patient‐specific human‐induced pluripotent stem cells (hiPSCs)‐differentiated myotubes and *OXA1L* knockout (KO) human immortalised skeletal muscle cell (IHSMC). Additionally, targeted depletion of *Oxa1l* in mouse skeletal muscle caused combined MRC defects as well as impaired muscle strength, mimicking the patients’ phenotypes. In OXA1L‐deficient cells, we further found higher ROS generation and increased nuclear factor kappa B (NF‐κB) signalling activity, potentially affecting myogenic programming and inducing cell apoptosis. These findings shed light on the pathophysiology of mitochondrial myopathy and identify novel intervention targets.

## 2 MATERIALS AND METHODS

### 2.1 Human subjects

The proband was a Chinese girl. Her clinical data for growth and development as well as related examinations such as magnetic resonance imaging (MRI), electromyography (EMG), abdomen radiograph, biochemical, metabolic and immunological examinations were obtained from electronic medical records. Follow‐up information was collected by patient's outpatient records. Blood samples were taken from four family members for further genetic testing and investigation.

### 2.2 Genetic testing

WES was conducted according to standardised procedures employing the xGen Exome Research Panel v1.0 hybridisation capture system (Integrated DNA Technologies) in accordance with the manufacturer's recommended guidelines. Subsequent sequencing of enriched DNA libraries was executed on an Illumina HiSeq 4000 sequencing system (San Diego, CA) using a paired‐end sequencing configuration with 150‐base pair read lengths. Raw sequencing data underwent alignment against the GRCH37/UCSC hg19 reference genome assembly, followed by variant identification through implementation of the GATK best practices pipeline, version 3.0. Functional annotation of genetic variants was subsequently performed using the SnpEff software suite (version 4.2). Variants exhibiting high population frequencies (defined as >1% allele frequency in either the gnomAD consortium or 1000 Genomes Project databases, or >5% frequency in an in‐house exome database containing > 6000 samples) were systematically excluded from further analysis. A multi‐tiered filtration strategy was implemented, incorporating autosomal recessive, autosomal dominant/de novo and X‐linked inheritance patterns. Validation of prioritised variants within the OXA1L locus was achieved through Sanger sequencing, with subsequent segregation analysis performed in pedigree members. Primer sequences utilised for PCR amplification are detailed in Table S1.

### 2.3 Generation of immortalised lymphoblastoid cell lines

Peripheral blood mononuclear cells (PBMCs) were purified through density gradient centrifugation using Ficoll‐Paque Plus (GE Healthcare, Boston, MA, USA). Briefly, after being mixed 1:1 with RPMI 1640 medium (Corning, Cambridge, MA, USA), the peripheral blood was stratified layering in sterile 15 mL conical tubes containing density gradient medium. Centrifugal processing was performed at 400*g* for 30 min under ambient conditions without brake, after which the interphase PBMC band was aspirated with precision. Harvested cells underwent two sequential washing cycles in RPMI 1640 medium through 5‐min centrifugations at 200×*g* under ambient conditions. For immortalisation procedures, PBMC suspensions were standardised to 2 × 10^6^ cells/mL in optimised culture medium comprising equal volumes of RPMI 1640 supplemented with 20% foetal bovine serum (FBS; Thermo Fisher Scientific, Waltham, MA) and Epstein‐Barr viral (EBV) stock, further supplemented with 2 µg/mL cyclosporin A (Meilunbio, Dalian, China). Primary cultures were maintained in a humidified 5% CO2 atmosphere at 37°C. Upon macroscopic observation of EBV‐induced cellular aggregation (typically occurring within 7 days post‐infection), partial medium replacement (50% volume) was conducted every 72–96 h using fresh growth medium. Subculture procedures were initiated weekly until establishment of stable proliferative patterns. Cryopreservation of immortalised lymphoblastoid cell lines (LCLs) was achieved through suspension in 90% FBS supplemented with 10% DMSO cryoprotectant (Sigma–Aldrich, St. Louis, MO, USA). Control lymphocyte specimens were obtained from an age‐matched healthy female donor under informed consent for research participation.

### 2.4 RT‐PCR analysis of the splicing variant

RT‐PCR was used to identify the alternative splicing patterns using the primers listed in Table S2. RNA extraction from LCLs and differentiated myotubes was performed using Trizol reagent (Thermo Fisher Scientific) as directed by the manufacturer, with subsequent quantification conducted via Nanodrop spectrophotometry. For cDNA synthesis, 1 µg of total RNA underwent reverse transcription using the PrimeScript™ RT reagent Kit with gDNA Eraser (TaKaRa Bio, Otsu, Japan). Subsequent amplification reactions were carried out with PrimeSTAR^®^ Max DNA Polymerase (TaKaRa Bio) under optimised thermocycling conditions. Amplification products were subjected to electrophoretic separation in 2% agarose gels containing ethidium bromide at 100 V for 40 min. For precise characterisation of splice variants, gel‐purified amplicons were analysed through Sanger sequencing.

### 2.5 Generation and culture of hiPSCs

To generate hiPSCs, a combination of three reprogramming non‐integrating episomal vectors (pCXLE‐hOCT3/4‐shp53‐F, pCXLE‐hSK and pCXLE‐hUL; 10 µg each) were electroporated into LCLs employing the Neon™ Transfection System (MPK10096; Thermo Fisher Scientific) following manufacturer's instruction. Following electroporation, cellular suspensions were resuspended in RPMI 1640 medium supplemented with 20% FBS, plated into mouse embryonic fibroblast feeder layers in six‐well culture plates. On the first day following electrotransformation, add 1 mL of mTeSR™ Plus medium (StemCell Technologies, Canada). Semi‐fluid changes were performed on day 2–6 post‐electrotransformation, with complete fluid changes beginning on day 7. After 3–4 weeks, the undifferentiated hiPSC colonies were picked, Accutase (Merck, USA) digested and expanded on Matrigel‐coated (Corning) plates in mTeSR™ Plus medium in a humidified incubator at 37°C, and 5% CO_2_. Immunostaining and quantitative real‐time PCR (qPCR) of pluripotency markers, and teratoma formation were used to validate the characteristics of hiPSCs. Used qPCR primers are described in Table S4.

### 2.6 Karyotype analysis

To evaluate the genetic stability of the reprogrammed hiPSC beyond passage 10, karyotyping was performed through conventional cytogenetic analysis. In brief, cellular synchronisation was achieved via 60‐min incubation with Colcemid (10 µg/mL; Thermo Fisher Scientific) under standard culture conditions (37°C, 5% CO₂). After dissociation with Accutase, cells underwent sequential processing: (1) hypotonic treatment with 0.075 M potassium chloride solution, (2) three‐cycle fixation in freshly prepared Carnoy's fixative and (3) controlled chromosomal spread preparation through standardised drop‐splash methodology on pre‐chilled microscope slides. Metaphase spreads were subsequently prepared and analysed using G‐banding to assess chromosomal stability.

### 2.7 Myotube differentiation of hiPSCs

hiPSCs were enzymatically dissociated to single‐cell suspensions and seeded at 4 × 10^4^ cells/cm^2^ density onto Matrigel‐coated six‐well plates (Corning) in mTeSR™ Plus media with 10 µM Y‐27632 (Selleck, Houston, TX, USA). After incubation at 37°C for 24 h, myogenic differentiation was initiated using the STEMdiff™ Myogenic Progenitor Kit (StemCell Technologies) through sequential media changes: STEMdiff™ Myogenic Progenitor Medium A for 2 days, Medium B for 2 days, and Medium C for 2 days, with daily medium replacements. Subsequently, cells were maintained in Medium D with daily medium refreshment until day 30 of differentiation. Resultant myogenic progenitor cells (MPCs) were expanded in MyoCult™‐SF Expansion Medium (StemCell Technologies), then cryopreserved for subsequent differentiation. To differentiate into myotubes, 8 × 10^4^ MPCs/well were seeded on fresh Matrigel substrates in MyoCult™‐SF Expansion Medium and change medium every other day. Upon reaching 95–100% confluence, differentiation was induced by switching to MyoCult™ Differentiation Medium (1.5 mL/well), maintained for 8 days with daily 50% medium replenishment. For the rescue experiment, MPCs were pretreated with 75 mM Mito‐TEMPO (MedChemExpress, New Jersey, USA) for 48 h prior to myotube induction, followed by a supplemental dose administered on day 5 post‐induction until cells were harvested for downstream assays.

### 2.8 KO and re‐expression of OXA1L

Clustered regularly interspaced short palindromic repeats (CRISPR)/Cas9 genomic editing technology was employed to generate *OXA1L* KO IHSMC. sgRNA sequences (Table S3) were designed using https://zlab.bio/guide‐design‐resources and then inserted into lentiCRISPR v2 vector. The successfully cloned sgRNA plasmids, psPAX2 packaging vector and pMD2G envelope vector were transfected into HEK293T cells at a 2:1 ratio using Lipofectamine 3000 (Thermo Fisher Scientific) to produce lentivirus over 3 days. IHSMC was infected with the harvested lentivirus for 48 h before being selected for 7 days in 0.6 µg/mL puromycin‐containing medium and submitted to western blotting analysis to validate OXA1L expression. For OXA1L re‐expression, OXA1L KO IHSMC were infected with FLAG‐tagged OXA1L wild type (WT) or variant (Cys207Phe, Cys207_Glu254del and Val388Alafs*15) plasmids (YouBio, Changsha, Hunan, China) encapsulated viral fluids for 48 h before being selected in 10 µg/mL blasticidin‐containing media for 7 days. Pt‐hiPSC differentiated MPCs were also infected with OXA1L WT plasmid‐enveloped virus for 48 h, followed by selection in medium containing 12.5 µg/mL blasticidin for 7 days.

### 2.9 Quantitative real‐time PCR analysis

RNA isolation was performed with Trizol reagent, followed by Nanodrop spectrophotometric quantification. gDNA Eraser treatment (42°C, 2 min) effectively eliminated genomic DNA contamination, after which reverse transcription was conducted under the following thermal cycling conditions: 37°C for cDNA synthesis (30 min) followed by enzyme inactivation at 85°C (15 s). Quantitative RCR analysis on the ViiA 7 platform (Applied Biosystems, USA) utilised TB Green® Premix Ex Taq™ (TaKaRa Bio) in 10 µL reaction system: 5 µL 2× TB Green Premix Ex Taq, 0.5 µL each forward/reverse primer (10 µM), 1 µL diluted cDNA template (1:10), 0.2 µL ROX II (50×), 2.8 µL nuclease‐free water. Expression values were analysed with Microsoft Excel 2016 (Microsoft, USA), normalised to the housekeeping gene β‐Actin expression. The primer sequences used are described in Table S4.

### 2.10 Immunofluorescence staining

Cellular immunofluorescence processing involved sequential treatments: fixation in 4% paraformaldehyde (Beyotime, China; 15 min, RT), permeabilisation using 0.3% Triton X‐100 (Merck; 10 min) and blocking with 10% goat serum (Gibco, USA; 60–90 min, RT). Primary antibody incubation proceeded overnight at 4°C, followed by 37°C exposure to Alexa Fluor‐conjugated secondary antibodies (594/488; 1–2 h) and DAPI counterstaining (37°C, 10 min) for nuclear visualisation. Fluorescence imaging was executed using a THUNDER Imager (Leica, Heerbrugg, Switzerland). The antibodies are described in Table S5.

### 2.11 Western blotting and blue native PAGE

The protein extracts from cell lysates and skeletal muscles for western blotting underwent homogenisation in RIPA buffer supplemented with protease/phosphatase inhibitors (MedChemExpress). For blue native PAGE (BN‐PAGE), proteins were extracted using Triton X‐100‐containing solubilisation buffer (50 mM NaCl; 50 mM Tris–Base; 2 mM 6‐aminohexanoic acid; 1 mM EDTA). Protein quantification employed a bicinchoninic acid protein assay kit (Takara Bio) as directed by the manufacturer. Electrophoretic separation was performed via 10% SDS‐PAGE or gradient BN‐PAGE (3.5–16%), followed by electrophoretic transfer to PVDF membranes (Merck Millipore, USA). Membranes were sequentially treated with: 5% non‐fat milk blocking (2 h, RT), primary antibody incubation (overnight, 4°C on a shaker), and HRP‐conjugated secondary antibody exposure (2 h, RT). Band intensity quantification utilised ImageJ (NIH, USA), with antibody specifications detailed in Table S6.

### 2.12 Evaluation of mitochondrial complex activities

Mitochondria fractions from patient‐derived myotubes and IHSMC cells were extracted to measure the enzymatic activities of MRC complexes, as previously described.^22^ Following homogenisation, mitochondrial proteins were extracted via three freeze–thaw cycles with liquid nitrogen. OXPHOS activities were quantified through substrate‐specific enzymatic reactions. CI: Rotenone‐sensitive NADH oxidation (340 nm). CII: 2,6‐Dichloroindolol reduction rate (600 nm). CIII: Reduced cytochrome *c* accumulation (550 nm). CIV: Oxidised cytochrome *c* depletion (550 nm). CV: NADH oxidation kinetics (340 nm). The complex activities of mouse skeletal muscles were determined with commercial assay kits (Solarbio, Beijing, China). Skeletal muscles underwent washing, homogenisation in isolation buffer and sequential centrifugation (600×*g*, 10 min; 11,000×*g*, 15 min; 4°C). Pelleted mitochondria were reconstituted in assay buffer, incubated with substrates and analysed at specified wavelengths per kit guidelines.

### 2.13 Mitochondrial respiration detection

Mitochondrial respiratory profiling in IHSMC was conducted using the Oxygraph‐2k respirometer (Oroboros, Austria). Following the assessment of baseline oxygen respiration in the absence of any additives, sequential additions of 2.5 µg/mL oligomycin (Sigma–Aldrich) and 0.25 µg/mL FCCP (Sigma–Aldrich) were performed to quantify phosphorylation‐linked respiration and maximal electron transport chain capacity. Mitochondrial respiratory kinetics in isolated mitochondria from murine gastrocnemius muscles were evaluated using Oxygen Consumption Rate Assay Kit (BestBio, Nanjing, China). 100 µL mitochondrial suspensions were dispensed into 96‐well plates and combined with 10 µL of BBoxiProbe™ R02, a fluorescent oxygen sensor. Following homogenisation, each well was overlaid with 100 µL of oxygen barrier solution. Real‐time oxygen utilisation was tracked fluorometrically (Ex/Em: 460/600 nm), with measurements recorded at 2–3 min intervals. Resultant kinetic curves were analysed to quantify mitochondrial respiratory activity.

### 2.14 Measurement of intracellular ATP level

Intracellular ATP quantification was performed using a commercial ATP Assay Kit (Beyotime) following manufacturer protocols. Cell suspensions underwent sequential processing: collection, washing and lysis using kit‐provided buffer. Lysates were centrifuged (13,800 ×*g*, 5 min, 4°C) to isolate supernatants for analysis. In 96‐well plates, 100 µL ATP detection reagent was equilibrated (3–5 min, RT) before adding 20 µL standards/samples. Luminescence signals were captured using a BioTek microplate reader after at least 2 s interval. Final ATP concentrations were normalised against both standard calibration curves and total protein content.

### 2.15 Detection of lactic acid

Lactic acid quantification was performed with a commercial assay system (Njjcbio, Nangjing, China) following manufacturer guidelines. Cells underwent PBS washing, collection and ultrasonic homogenisation. Processed samples were centrifuged (600 ×*g*, 10 min, 4°C) to obtain clarified supernatants. Subsequently, the supernatant from cell lysates or mouse plasma was aliquoted into 96‐well plates, sequentially mixed with enzyme working solution and chromogenic reagent, respectively. Following 10‐min incubation at 37°C, reactions were terminated prior to spectrophotometric analysis at 530 nm. Cellular lactic acid levels were normalised to the total protein concentration to ensure accurate quantification.

### 2.16 Transgenic mice

All mouse studies complied with ethical standards approved by Xinhua Hospital's Institutional Animal Care Committee (School of Medicine, Shanghai Jiao Tong University). The *Oxa1l* gene is located on mouse chromosome 14 and contains 10 coding exons with the ATG start codon in Exon 1. Exon 3 is selected as the conditional KO (cKO) region with 2 loxp sites inserted in introns 2 and 3 (Figure S6A), enabling Cre‐dependent frameshift mutation upon Exon 3 deletion. The Cas9, gRNA and donor vector were co‐injected into fertilised eggs for cKO mouse production. Target gRNA sequences are described in Table S7. PCR was performed with primers flanking the loxP locus to identify the WT (309 bp) and floxed (376 bp) alleles. The primer sequences were as follows: forward primer, AGTGGTGGTAAGCCAATAAAGTGA; reverse primer, GCTCTGTTACTGACTACATCCCAA. Given embryonic mortality of *Oxa1l* ^−/−^, skeletal muscle‐specific *Oxa1l* cKO embryos (*Oxa1l^f/f, ACTA1‐Cre^*) were generated through crossbreeding with tamoxifen inducible *ACTA1*‐CreEsr1 mice.^23^ This system utilises a modified oestrogen receptor ligand‐binding domain that requires tamoxifen activation for Cre recombinase functionality. The presence of the ACTA1‐CreEsr1 transgene was validated by amplifying a characteristic ∼1100 base pair product with the following primers: forward primer, CGAGCCGAGAGTAGCAGTTGTA; reverse primer, AGGTGGACCTGATCATGGAG. For Cre activation, 8–12 week‐old mice received daily intraperitoneal injections (150 mg/kg BW) of tamoxifen (Sigma–Aldrich) dissolved in corn oil (10 mg/mL) for 5 consecutive days. In the rescue experiment, 1 mg/kg Mito‐TEMPO and 150 mg/kg tamoxifen was injected intraperitoneally daily for 5 consecutive days. Age and sex matched *Oxa1l^f/f^* mice were used as controls and received the same tamoxifen dose. Skeletal muscle‐specific deletion of *Oxa1l* was validated by amplifying a 458 bp product resulting from exon 3 excision in the *Oxa1l* gene (forward primer, AGTGGTGGTAAGCCAATAAAGTGA; reverse primer, AGTGGCATTAGGAGGTGTGTGGC). All animals were maintained under standardised husbandry conditions (12‐h photocycle, free access to food/ water).

### 2.17 Assessment of NAD^+^/NADH

NAD^+^/NADH quantification was performed according to the manufacturer's protocol (Beyotime) using a WST‐8‐based detection system. Gastrocnemius tissue samples (10 mg) were homogenised in 200 µL of pre‐cooled NAD^+^/NADH lysis buffer and centrifuged (12,000×*g*, 10 min, 4°C). Aliquots (50 µL) of supernatant underwent denaturation (60°C, 30 min) followed by centrifugation (10,000×*g*, 5 min, 4°C). Processed supernatants (20 µL) were incubated in 96‐well plates under dark conditions (37°C, 10 min). Following addition of 10 µL chromogenic substrate and thorough mixing, enzymatic reactions proceeded (37°C, 20 min, under dark). Spectrophotometric analysis at 450 nm enabled calculation of NAD^+^/NADH ratios through differential absorbance measurements.

### 2.18 Measurement of creatine kinase concentrations

Creatine kinase (CK) levels in mouse plasma were determined using Amplex Red Creatine Kinase Assay Kit (Beyotime) following manufacturer protocols. Aliquots (20 µL) of controls, standards or samples were combined with 80 µL of Amplex Red working solution in microplate wells and incubated for 2 min at 37°C away from light. Baseline fluorescence (RFU1) was measured at 560/590 nm excitation/emission wavelengths. Following 70‐min incubation at 37°C, endpoint measurements (RFU2) were obtained under identical spectral conditions. ΔRFU values (RFU2–RFU1) were applied to the calibration curve for enzymatic ADP generation quantification during the reaction period.

### 2.19 Detection of citric acid

Citric acid concentrations in mouse plasma were quantified using a commercial colorimetric assay kit (Elabscience, Wuhan, China) following standardised procedures. 30 µL aliquots of standards/samples were combined with 210 µL extraction buffer, 30 µL reducing solution and 30 µL chromogen in microcentrifuge tubes. After 30‐min room temperature incubation, 200 µL supernatant was transferred to 96‐well plates for spectrophotometric analysis at 545 nm.

### 2.20 Assessment of pyruvic acid

Pyruvic acid levels in mouse plasma were determined using a pyruvic acid colorimetric assay kit (Elabscience). Each 15 µL sample/standard received 50 µL chromogenic reagent in microplate wells, followed by 10s vortex mixing and 10 min incubation at 37°C. Subsequently, 150 µL alkaline solution was added to all wells, followed by brief vortexing and 5‐min room temperature incubation prior to absorbance measurement at 505 nm.

### 2.21 Open‐field test

The open‐field test was conducted on *Oxa1l* ^f/f^ (*n* = 10) and *Oxa1l^f/f, ACTA1‐Cre^* (*n* = 11) mice aged at 3–4 months. Mice were positioned centrally in a 50 × 50 × 50 cm^3^ container for 10‐min free exploration. The mouse movement tracks were captured using Monitor software, and the total distance was used to assess the mice's basal locomotor ability.

### 2.22 Balance beam test

The balance beam test was carried out on *Oxa1l* ^f/f^ (*n* = 10) and *Oxa1l^f/f, ACTA1‐Cre^* (*n* = 11) mice aged at 3–4 months. The beam utilised in this experiment was 100 cm long, 1 cm wide and 80 cm above the ground, with a home cage at one end. For 2 days, each mouse was trained to perform the task on three times. On day 3, the time of mice passing through the balance beam and the number of foot slips within 60 s were recorded by video.

### 2.23 Grip strength measurement

The grip strength quantification employed a digital dynamometer (Yiyan Technology, Shandong, China). Mice were placed horizontally on a rigid grid connected with a digital dynamometer. Each mouse's tail was lifted and dragged backwards gently until the mouse released the net. The measurement was conducted for 3 consecutive days, and the average of the peak force (g) of three trials was calculated.

### 2.24 The rotated test

Motor function was evaluated with DigBehv‐RRPM Rota‐Rod test system (Jiliang software technology, Shanghai, China). After the mice were acclimatised to the rod for 1 min, the speed of the rod steadily accelerated from 5 to 40 rpm within 5 min. During the procedure, the latency to fall was recorded. All animals were subjected to experiments for 5 consecutive days, three trials each day with a 10‐min rest in between. Data were presented as the mean of three tests.

### 2.25 Modified Gomori trichrome staining

Fresh gastrocnemius samples were cryopreserved in isopentane and sectioned into 10‐µm slices. Sections underwent staining with Weigert's iron haematoxylin for 5 min, then rinsed under running water for 2 min before immersing in tap water for 10 min to achieve bluing. After three washes in distilled water (2 min each), slices underwent 40‐min Gomori working solution incubation, followed by a 2 min tap water rinse. Subsequently, sections were incubated with the Gomori differentiation working solution for 60 s before being washed with running water for another 1–2 min. Dehydration comprised 10‐s 95% ethanol immersion followed by three 10 s absolute ethanol treatments. Finally, sections were xylene‐cleared (3 × 1 min) and resin‐mounted for microscopic analysis.

### 2.26 COX/SDH double staining

COX/SDH double staining was carried out on mouse gastrocnemius tissue using the COX‐SDH staining kit (Solarbio) as directed. Frozen tissue sections (10 µm) underwent sequential processing: 2 min COX clearing, 40 min dark‐phase COX staining (37°C) and triple 1‐min clearing cycles. Subsequently, sections underwent NBT substrate incubation at 37°C for 20 min, followed by two 1‐min washes with distilled water. After dehydration through a series of ethanol solutions, sections underwent xylene clearance and resin embedding for imaging.

### 2.27 Transmission electron microscopy

Gastrocnemius muscles excised from *Oxa1l^f/f^* or *Oxa1l^f/f, ACTA1‐Cre^* mice underwent primary fixation in 2.5% glutaraldehyde (4°C), followed by 1% OsO4 post‐fixation (2 h, RT). The skeletal muscles were sequentially dehydrated through graded ethanol/acetone series, embedded in EMBed 812 and sliced into 60 nm sections. Ultrathin sections were dual‐stained with uranium acetate/lead citrate prior to transmission electron microscope imaging (HT7800; HITACHI).

### 2.28 RNA‐sequencing

Total RNA samples from cultivated IHSMC and differentiated myotubes with three biological replicates were prepared using the RNeasy Mini Kit (Qiagen, Hilden, Germany). RNA integrity and quantity were validated via Bioanalyzer 2100 (Agilent, CA, USA). Qualified libraries underwent paired‐end sequencing on Illumina NovaSeq 6000. Clean reads were aligned to reference genomes using Hisat2 (v2.0.5). DESeq2 R (1.20.0) identified differentially expressed genes with padj ≤ 0.05 and |log2 (foldchange)| ≥ 1. Functional enrichment analysis employed clusterProfiler (v3.8.1) for gene ontology (GO)/KEGG terms, while gene set enrichment analysis (GSEA) utilised the Broad Institute's framework. Alternative splicing events were analysed using the rMATS (4.1.0) software, with ontology similarity assessed through simplifyEnrichment.^24^

### 2.29 Determination of intracellular and mitochondrial ROS generation

Intracellular ROS levels were quantified using a commercial ROS assay kit (Solarbio). Cells were collected, washed twice with 1× PBS (Corning), and then incubated with 10 µM DCFH‐DA (37°C, 30 min, dark), following by twice PBS rinses. Fluorescence intensity was measured spectrofluorometrically (BioTek) at 488/525 nm excitation/emission. For mitochondrial ROS, 5 × 105 cells were processed through MitoSOX Red staining (Beyotime). PBS‐washed samples received 5 µM probe (37°C, 30 min, dark), centrifuged (600×*g*, 3 min, 4°C) and rewashed. Fluorescence detection occurred at 510/580 nm excitation/emission using a fluorospectrophotometer (BioTek).

### 2.30 ROS detection of skeletal muscle

ROS levels in mouse skeletal muscle tissues were quantified with a tissue ROS detection kit (BestBio). 50 mg of fresh gastrocnemius muscles were homogenised in 1 mL buffer A and centrifuged (13,800 ×*g* , 10 min, 4°C). Aliquots (190 µL) were combined with BBoxiProbe O12 (10 µL) in microplates and dark‐incubated (37°C, 30 min). Fluorescence readings were recorded (excitation 488 nm/emission 530 nm) using a BioTek instrument, normalised to protein concentration for comparative analysis.

### 2.31 Detection of SOD activities

SOD activities were evaluated using a WST‐8‐based kit (Beyotime) as directed by the manufacturer. Briefly, 20 µL lysate supernatant was mixed with 160 µL WST‐8/enzyme working buffer in microplates. Reaction initiator (20 µL) was added before 37°C incubation (30 min). Absorbance at 450 nm was quantified using a BioTek microplate reader.

### 2.32 Measurement of TNF‐α levels

TNF‐α levels in IHSMCs and differentiated myotubes were determined using ELISA Kits (Jiancheng, Jiangsu, China) per manufacturer guidelines. Cell suspensions (1 × 10^6^ cells/mL PBS) underwent three freeze–thaw cycles, followed by centrifugation (900 ×*g*, 20 min). Supernatants (50 µL/well) and standards were incubated with biotinylated antibody solution (50 µL/well) at 37°C for 30 min. After washing five times, HRP conjugate (50 µL/well) was added and incubated (37°C, 30 min). After repeated washing, chromogenic substrates A/B (50 µL each) were added for 10 min incubation (37°C, dark). Reactions were terminated with stop solution (50 µL/well), and absorbance at 450 nm was recorded within 10 min using ELISAcalc software for standard curve‐based quantification.

### 2.33 TUNEL staining

Apoptosis levels were evaluated using a commercial terminal deoxynucleotidyl transferase‐mediated dUTP‐biotin nick end labelling (TUNEL) kit (Beyotime). Following PBS rinsing, samples were fixed in 4% paraformaldehyde (30 min, RT), permeabilised with 0.3% Triton X‐100 (5 min, RT), then incubated with the TUNEL reaction mixture (1 h, 37°C, dark). TUNEL‐positive cells were photographed via a fluorescence microscope (Leica) with nuclei counterstained with DAPI.

### 2.34 Statistical analysis

All data were shown as mean ± SEM. Statistical processing was conducted using GraphPad Prism 8.3.0 and SPSS Statistics 25.0 software (IBM, Armonk, NY). Data normality was verified through Shapiro–Wilk test, while variance homogeneity was evaluated using Levene's test. Inter‐group comparisons employed either parametric two‐tailed Student's *t*‐tests (for data meeting normality and homogeneity of variance assumptions) or non‐parametric Mann–Whitney *U* test (for non‐parametric distributions). Multi‐group parametric analyses utilised one‐way ANOVA with Bonferroni post hoc correction, whereas non‐parametric datasets were analysed via Kruskal–Wallis test followed by Dunn's multiple comparisons test. Significance thresholds were defined as *p < .05, **p < .01, and ***p < .001.

## 3 RESULTS

### 3.1 Clinical and genetic features of the proband with bi‐allelic *OXA1L* variants

The proband was an 11‐year‐old Chinese girl, the second child of asymptomatic non‐consanguineous parents; her older brother did not exhibit any comparable symptoms. She was born full‐term without perinatal complications. Motor development milestones were initially normal, with independent walking achieved at 18 months and ability to climb stairs independently before the age of 3 years. Progressive motor regression emerged post‐3 years old, characterised by lower‐extremity weakness, unsteady gait, generalised hypotonia and muscle atrophy. EMG tests revealed muscular and peripheral nerve damage. Physical examination revealed reduced bilateral tendon reflexes and absence of cerebellar signs. Neurological examination revealed no ocular motility deficits and preserved bilateral deep and superficial sensations in the patient. Biochemical investigations demonstrated elevated levels of blood lactate (2.6 mmol/L; control levels, 0.5–1.6 mmol/L), lactate dehydrogenase (262 U/L; control levels, 106–211 U/L), ammonia (47 µmol/L, control levels, 9–30 µmol/L) and uric acid (498 µmol/L, control levels, 155–357 µmol/L), while CK level (74 U/L; reference: 26–192 U/L) and haemoglobin level (123 g/L, reference values 110–160 g/L) were normal. Other relevant examinations, including cranial and pituitary MRI, abdominal radiograph, electrocardiogram, biochemical, metabolic and immunological examinations revealed nothing unusual. Upper extremity weakness, wrist dorsiflexion and blepharoptosis began to occur when she was 7 years old. Currently, the subject has sufficient cognitive and language abilities, but exhibits diffuse muscle weakness (upper extremities: bilateral proximal 4/5, distal 2/5: lower extremities: right 3/5, left 2/5: right 3/5, left 2/5), generalised hypotonia and obesity (height 140 cm, weight 57 kg, BMI 29 kg/m^2^), with complete loss of independent standing/walking capacity without assistance. Although histopathological confirmation is lacking, the integration of the patient's clinical and biochemical evidence meets diagnostic criteria for probable mitochondrial disease.^25^, ^26^

**FIGURE 1:**
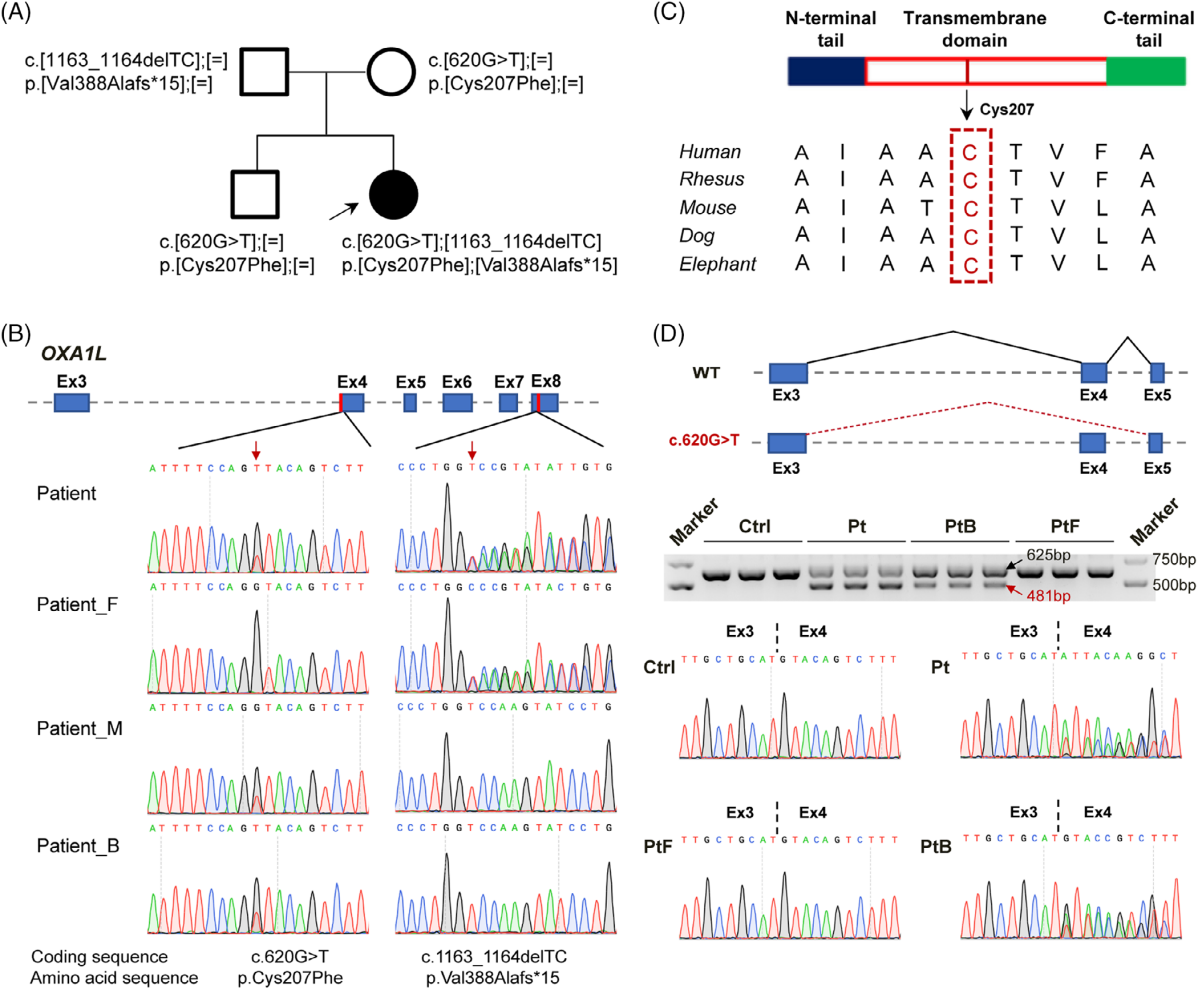
Genetic characteristics of *OXA1L* variants identified in the patient. (A) Family pedigree shows the proband (black arrow) harbours bi‐allelic *OXA1L* variants. (B) The bi‐allelic *OXA1L* variants were confirmed to be present on the *OXA1L* gene in the proband by genomic PCR and Sanger sequencing. (C) Schematic showing the OXA1L human Cys207 residue (in red) is highly conserved. (D) RT‐PCR analysis of the changed splicing patterns of *OXA1L* gene in patient‐derived LCLs.

To investigate possible causal genetic variants, WES was performed on the individual, as well as her unaffected parents. Bi‐allelic variants of *OXA1L* (GenBank: NM_005015.3: c.620G>T, p.[Cys207Phe] and c.1163_1164del, p.[Val388Alafs*15]) was identified to be inherited from her healthy parents and segregated with disease (Figure 1A,B). No other clinically significant detrimental variants were detected in this individual by WES and mtDNA sequencing. The frameshift variant c.1163_1164del was absent in general population database including ExAC and gnomAD, and has not been described previously in HGMD or ClinVar, indicating that it is a novel variant. In the ExAC and gnomAD databases, the allele frequency of c.620G>T variant is 0.000824 and 0.000398%, respectively, with no homozygotes recorded. The Cys207 residue affected by this variant, is highly conserved (score in phastCons: 1.0) among diverse species (Figure 1C). Several in silico prediction tools, including SIFT (score = 0.037), Mutation Taster (score = 1.0) and CADD (score = 24.9) predicted that the c.620G>T variant was damaging. Since this variant is located at the first nucleotide of exon 4, in addition to resulting in missense substitution, it is also predicted to disrupt splicing. RT‐PCR analysis of patient‐ and elder brother‐derived immortalised LCLs carrying the c.620G > T variant revealed co‐existing WT transcripts (EX4‐included) and aberrant transcripts (EX4‐skipped) (Figure 1D). This partial splicing dysregulation suggests a leaky pathogenic mechanism, where residual normal splicing occurs alongside variant‐induced defective processing. Sanger sequencing further confirmed this variant leads to exon 4 skipping (c.620_763del, p.[Cys207_Glu254del]) or the p.Cys207Phe amino acid substitution (Figure 1D), mirroring previous molecular finding in affected individual.^21^ Above findings suggested that the bi‐allelic variants c.620G>T and c.1163_1164del in *OXA1L* gene may be pathogenic genetic factors responsible for the mitochondrial myopathy in this individual.

### 3.2 OXA1L deficiency decreased the steady‐state levels and assembly of MRC complexes

To verify the variants indeed affect OXA1L expression, we first conducted quantitative real‐time PCR (qRT‐PCR) using mRNA extracted from LCLs derived from the patient (Pt), her unaffected father (PtF) and brother (PtB), and an age‐matched healthy control (Ctrl). The patient's relative *OXA1L* mRNA level was significantly lower than that of controls (Ctrl, PtB and PtF)‐derived LCLs (Figure S1A). Meanwhile, compared with the normal Ctrl‐LCLs, the relative *OXA1L* mRNA levels was both decreased in PtF‐ and PtB‐LCLs, each carrying a single heterozygous variant, while the OXA1L protein levels revealed no significant difference (Figure S1A–C). However, patient‐derived LCLs showed remarkably lower OXA1L protein level as compared with controls (Figure S1B,C).

To clarify the impact of *OXA1L* variants on MRC complexes, mRNA and protein levels of CI–CV subunits were detected respectively. The relative mRNA levels of SDHD (CII), UQCRC2 (CIII) and ATP5A (CV) in Pt‐derived LCLs were considerably declined compared with Ctrl, whereas the expression of the remaining MRC complex related genes, including NDUFA13 and NDUFA9 (CI), SDHB (CII), UQCRB (CIII), COX10 and COX6B1 (CIV), and ATP5B (CV) showed no major differences in patient as compared with controls (Figure S1D–H). However, no alteration in the protein content of MRC complexes was observed (Figure S1I).

**FIGURE 2:**
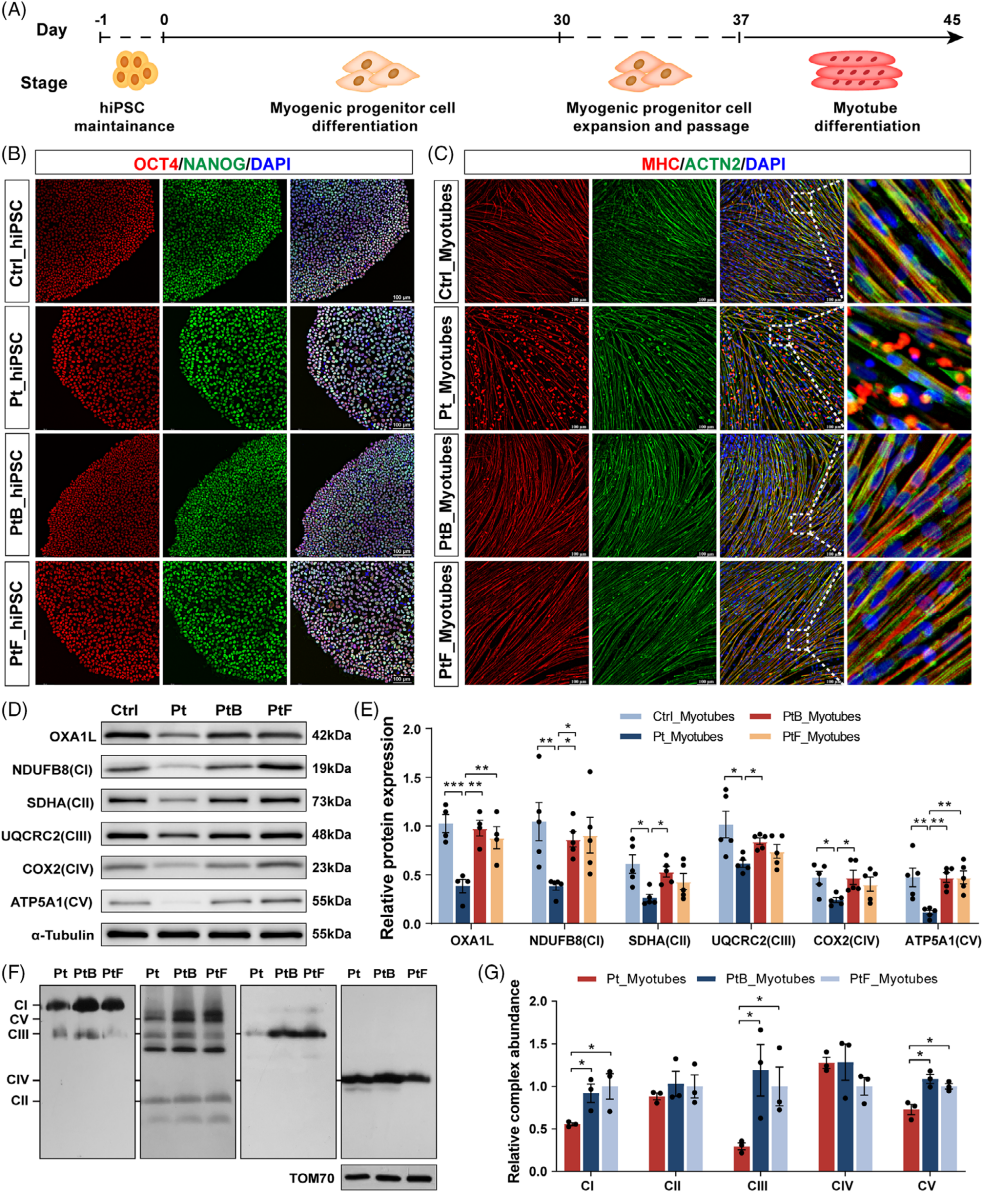
Impaired phenotypic and complex subunits in patient‐derived myotubes. (A) Schematic outline illustrating the critical differentiation procedure from the hiPSCs into myotubes. (B) Representative fluorescence images of hiPSC clones expressing pluripotency markers OCT4 (red) and NANOG (green) with nuclear staining (DNA, blue). Scale bar = 100 µm. (C) Representative staining of myosin heavy chain (MHC, red) and sarcomeric α‐actinin (ACTN2, green) in myotubes induced from hiPSCs. Scale bar = 100 µm. (D and E) Images and statistics of OXA1L and MRC subunits measured by western blotting analysis in myotubes (*n* ≥ 4). Data are presented as mean ± SEM. *p < .05, **p < .01, ***p < .001, one‐way ANOVA with Bonferroni post hoc test for OXA1L and ATP5A1, and Kruskal–Wallis test with Dunn's multiple comparisons test for NDUFB8, SDHA, UQCRC2 and COX2. (F and G) Images and statistics of MRC assembly analysed by BN‐PAGE in myotubes (*n* = 3). Data are presented as mean ± SEM. *p < .05, unpaired *t*‐test.

Since the patient's dominant phenotype is focused in skeletal muscle, patient‐ and controls‐ (Ctrl, PtB and PtF) LCLs were reprogrammed into hiPSCs to better investigate the influence of variants on skeletal muscle. Myotubes were differentiated from these hiPSCs (Figure 2A) after identifying pluripotency by marker expression (Figure S2A), staining (Figures 2B and S2B) and teratoma formation (Figure S2C), and validation of normal karyotypic integrity (Figure S2D). The morphology of patient‐derived MPC was comparable to that of controls (Figure S3A,B), but increased dead cells began to be observed from day 5 of MPC induction into myotubes (Figure S3C). Immunofluorescence staining showed normal expression of MHC and ACTN2, but fragmented myofilaments were substantially higher in patient myotubes than in controls (Figure 2C).

Consistent with findings in LCLs, patient‐ and elder brother‐derived myotubes harbouring the c.620G > T variant exhibited coexistence of WT (EX4‐included) and aberrant (EX4‐skipped) transcripts (Figure S4). The relative OXA1L protein level in patient‐induced myotubes was considerably declined compared with controls (Figure 2D,E). Notably, protein levels of NDUFB8 (CI), SDHA (CII), UQCRC2 (CIII), COX2 (CIV) and ATP5A1 (CV) were strongly reduced in patient myotubes (Figure 2D,E). Western blotting analysis also revealed reductions of mtDNA‐encoded CIII (CYTB) and CV (ATP8) subunits in patient‐derived myotubes (Figure S5A,B). Furthermore, blue native PAGE (BN‐PAGE) assessment identified assembly defects of CI, CIII and CV in patient‐derived myotubes (Figure 2F,G).

**FIGURE 3:**
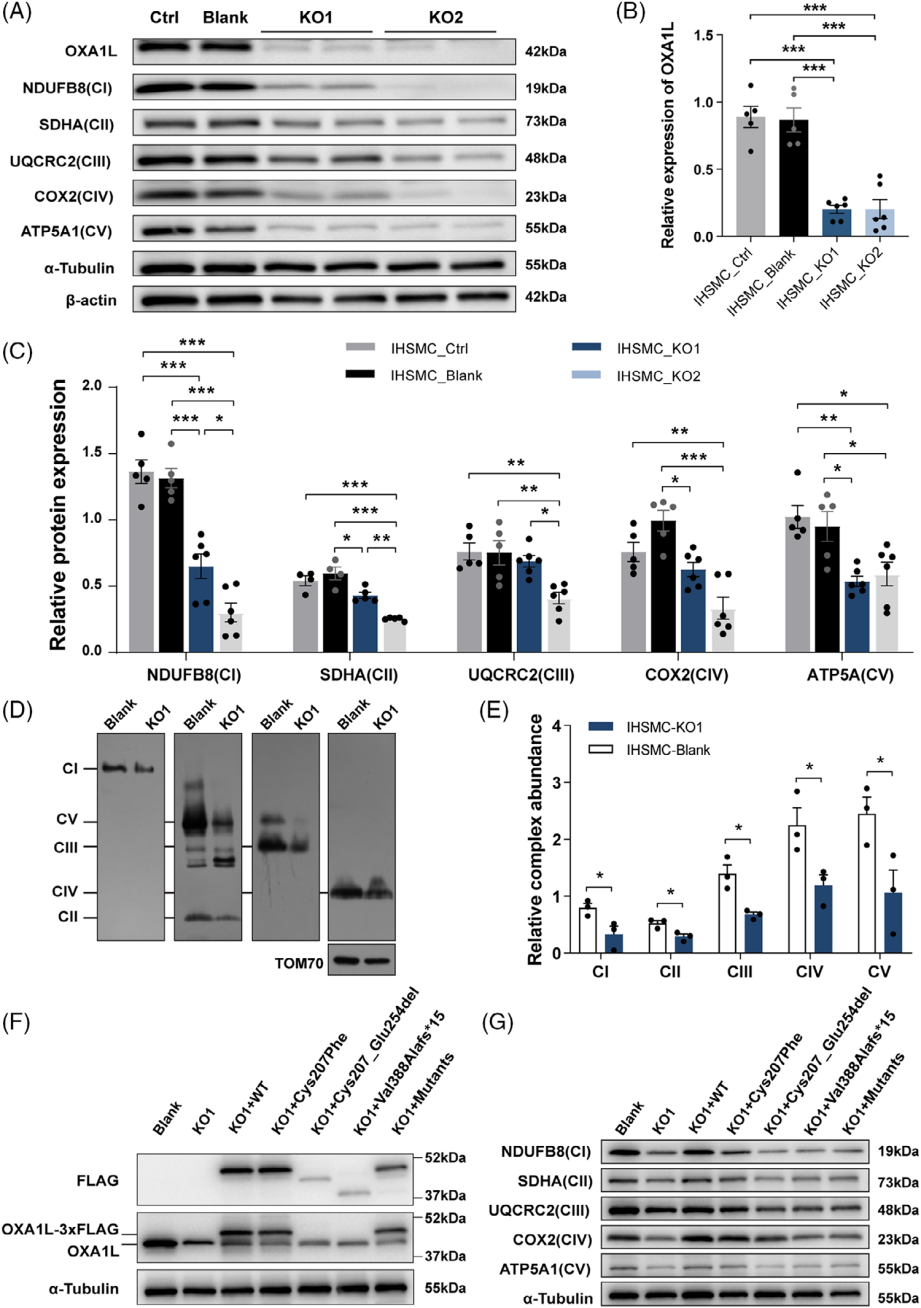
*OXA1L* depletion affects the expression of MRC complexes. (A) Western blotting analysis of OXA1L and MRC complex subunits in controls (Ctrl, Blank) and knockout (KO1, KO2) IHSMC. Ctrl: untransfected IHSMC; Blank: IHSMC transfected with empty vector. (B) Statistics of OXA1L measured by western blotting analysis. Data are shown as mean ± SEM (*n* ≥ 5 replicates; ***p < .001 by one‐way ANOVA). (C) Statistics of MRC subunits measured by western blotting analysis. Data are shown as mean ± SEM (*n* ≥ 4 replicates; *p < .05, **p < .01, ***p < .001 by one‐way ANOVA). (D and E) Representative images (D) and statistics (E) of MRC assembly analysed by BN‐PAGE in IHSMC. Data are shown as mean ± SEM (*n* = 3 replicates; *p < .05, unpaired *t*‐test). (F) The expression patterns of both endogenous OXA1L and exogenous FLAG in *OXA1L*‐KO1 IHSMC transfected with 3× FLAG‐tagged OXA1L constructs, including wild type (WT), Cys207Phe, Cys207_Glu254del, Val388Alafs*15 and mixed mutants. The endogenous OXA1L expression was detected independently, while the exogenously expressed proteins were identified through their 3× FLAG tag. (G) The expression of MRC subunits measured by western blotting in KO1 IHSMC transfected with WT and mutant plasmids.

To eliminate the impacts of genetic heterogeneity and differentiation variation between distinct individual‐derived hiPSCs, *OXA1L* KO IHSMC were generated using CRISPR/Cas9 technology. Two KO IHSMC lines (KO1 and KO2) targeting different loci in exon 3 of *OXA1L* gene were constructed (Figure S6A). Although not completely absent, OXA1L protein levels were demonstrated to be greatly decreased in both KO IHSMC lines (Figure 3A,B). Targeted *OXA1L* depletion in IHSMC caused defects in steady‐state levels of CI–CV, consistent with results in patient‐myotubes (Figure 3A,C). In addition, the mtDNA‐encoded CI (ND2), CIII (CYTB), CIV (COX1) and CV (ATP8) subunits were reduced in KO IHSMC lines (Figure S6B,C). The assembly defects of CI‐CV were also identified in KO IHSMC by BN‐PAGE analysis (Figure 3D,E).

Overexpression of WT OXA1L in patient‐derived myotubes rescued OXA1L deficiency (Figure S5C) as well as MRC defects (Figure S5D). Crucially, expressing WT OXA1L instead of the Cys207_Glu254del and Val388Alafs*15 mutant forms rescued the OXA1L deficiency (Figure 3F) and MRC defects (Figure 3G) in OXA1L depleted IHSMC. Overexpression of the Cys207Phe missense mutant was also shown to partially rescue MRC deficiency in *OXA1L*‐KO1 IHSMC. The above results demonstrate the pathogenicity of *OXA1L* variants in disrupting the biosynthesis of MRC complexes.

### 3.3 OXA1L deficiency impaired mitochondrial OXPHOS function

**FIGURE 4:**
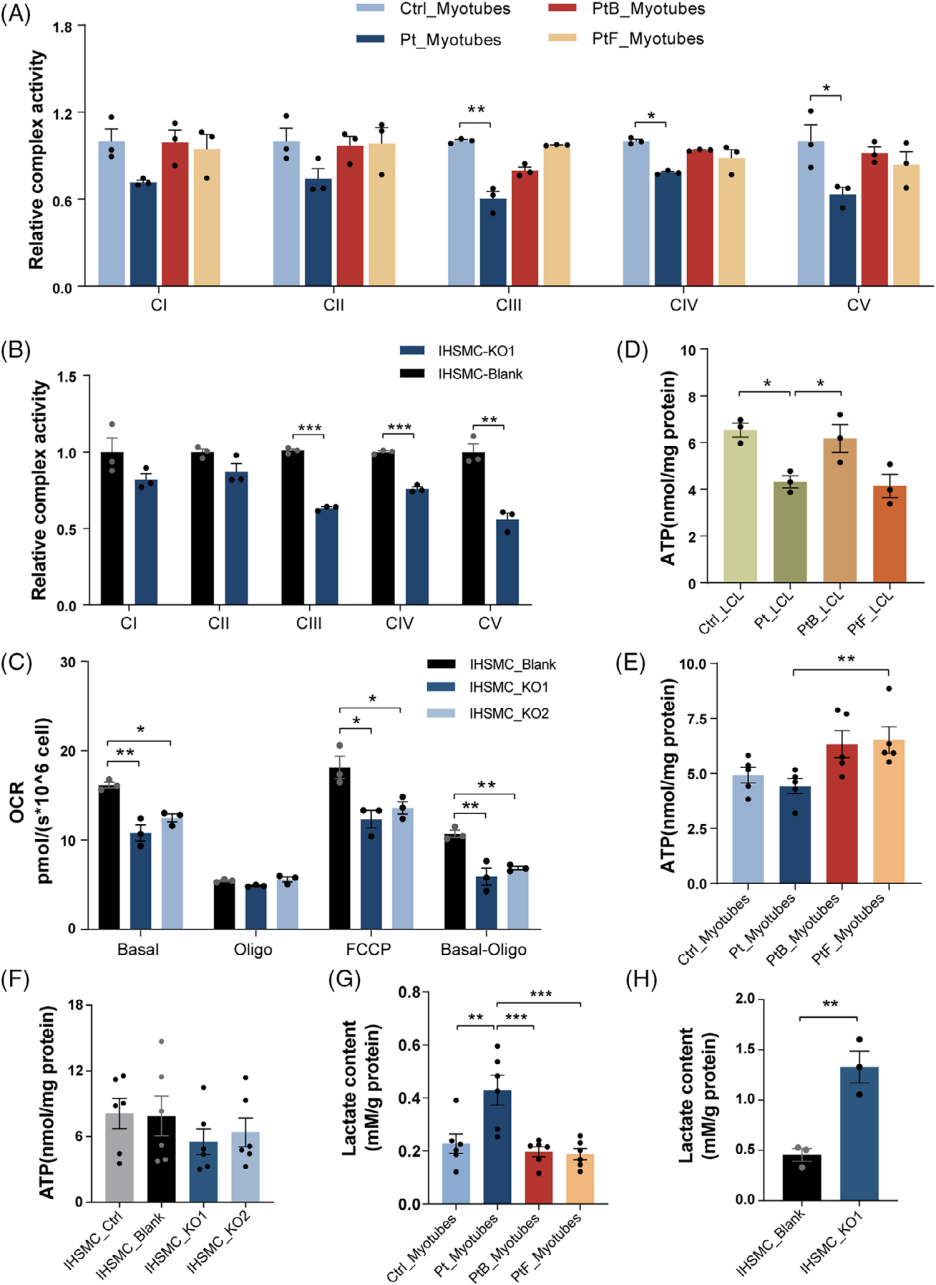
*OXA1L* deficiency impairs mitochondrial OXPHOS function. (A) The relative activities of OXPHOS complexes in patient (Pt) and controls (Ctrl, PtF, PtB)‐derived myotubes (*n* = 3; *p < .05, **p < .01; one‐way ANOVA with Bonferroni post hoc test for CI and CV, and Kruskal–Wallis test with Dunn's multiple comparisons test for CII, CIII and CIV). (B) The relative OXPHOS activities of the *OXA1L* KO1 IHSMC, compared with Blank IHSMC (*n* = 3; **p < .01, ***p < .001; unpaired *t*‐test for CI, CIII, CIV and CIV, and Mann–Whitney *U* test for CII). (C) Basal oxygen consumption rate in blank and knockout (KO1, KO2) IHSMC and response to injection of oligomycin and FCCP (*n* = 3; *p < .05, **p < .01; one‐way ANOVA with Bonferroni post hoc test). (D) Relative ATP content in patient (Pt) and controls (Ctrl, PtF, PtB)‐derived LCL (*n* = 3; *p < .05; one‐way ANOVA with Bonferroni post hoc test). (E) Relative ATP content in patient (Pt) and controls (Ctrl, PtF, PtB)‐derived myotubes (*n* = 5; **p < .01; Kruskal–Wallis test with Dunn's multiple comparisons test). (F) Relative ATP levels in *OXA1L* depleted IHSMC (*n* = 6; one‐way ANOVA with Bonferroni post hoc test). (G) Relative lactate concentrations determined in patient (Pt) and controls (Ctrl, PtF, PtB)‐derived myotubes (*n* = 6; **p < .01, ***p < .001; one‐way ANOVA with Bonferroni post hoc test). (H) Relative lactate concentrations measured in *OXA1L* depleted IHSMC (*n* = 3; **p < .01; unpaired *t*‐test). All data are presented as mean ± SEM.

To determine whether OXA1L deficiency altered mitochondrial OXPHOS function, we initially assessed MRC enzyme activity in patient‐myotubes and *OXA1L* KO IHSMC. MRC activity measurements revealed prominent reductions in the activities of CIII, CIV and CV in patient‐derived myotubes (Figure 4A) and *OXA1L* KO IHSMC (Figure 4B) compared with respective controls. We then analysed the oxygen consumption rate (OCR) in *OXA1L* KO IHSMC to assess the alterations in mitochondrial respiratory capacity (Figure 4C). The basal respirations were significantly reduced in both *OXA1L* KO IHSMC (Figure 4D). The maximal oxygen consumption and ATP‐coupled respiration were then evaluated after addition of the chemical uncoupler FCCP or oligomycin, the inhibitor of ATP synthase. Decreased maximal respiration and ATP‐linked respiration was detected in both *OXA1L* KO IHSMC lines compared with those in control (Figure 4D). Direct intercellular ATP generation assay revealed a considerably decrease in both patient‐derived LCL and myotubes compared with controls (Figure 4D,E). Whereas, no major difference in ATP production was detected between *OXA1L* KO IHSMC and controls (Figure 4F). Lactate levels were increased both in patient‐myotubes and *OXA1L* KO IHSMC as compared with respective controls (Figure 4G,H). Collectively, our results indicated that OXA1L deficiency impairs mitochondrial OXPHOS function.

### 3.4 *Oxa1l* depletion affected MRC complexes and motor abilities of mouse

**FIGURE 5:**
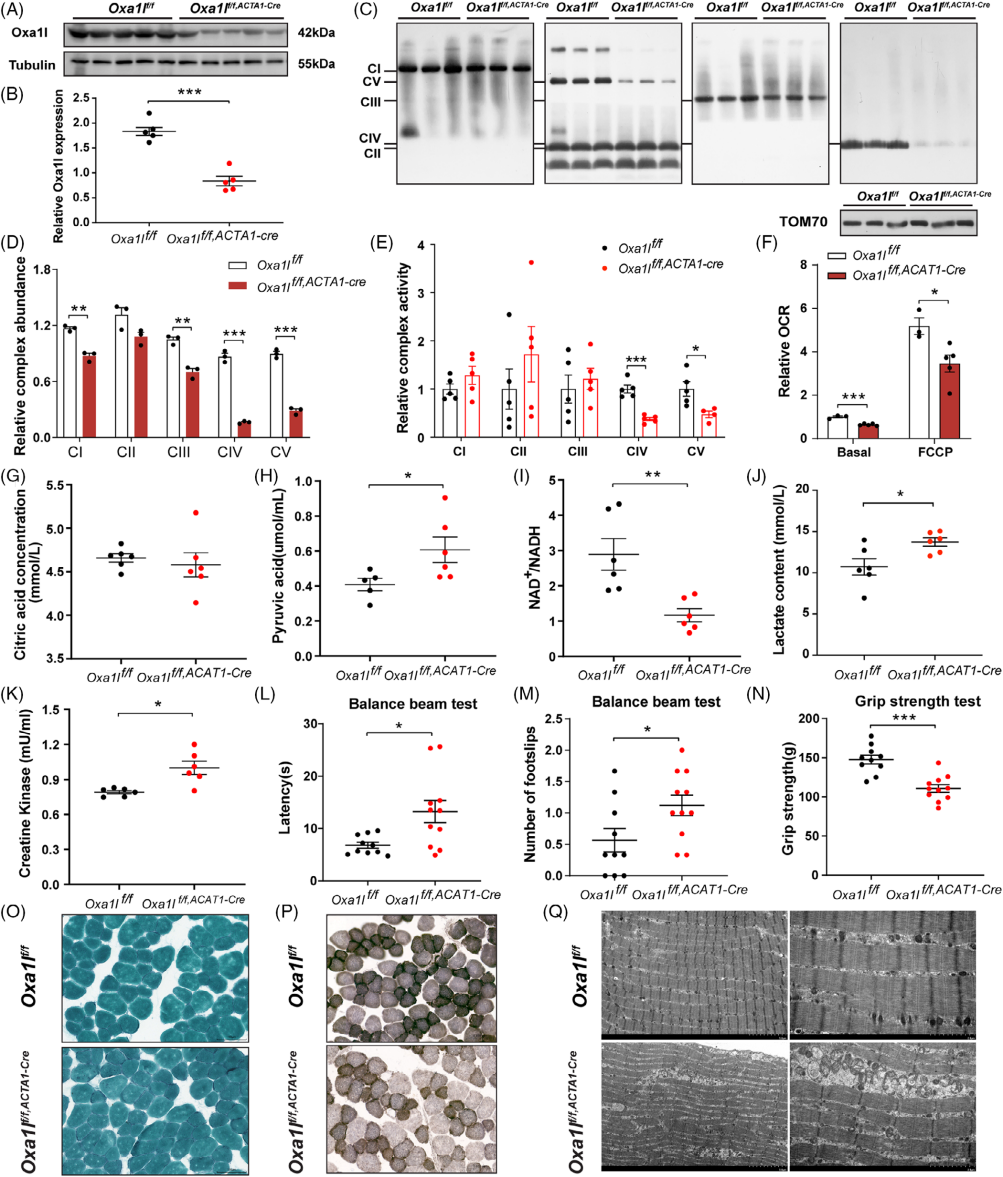
The behavioural, pathological and functional changes in *Oxa1l* cKO mice. (A and B) Western blotting analysis of Oxa1l expression in *Oxa1l* cKO mice and littermate control mice (*n* = 5 mice per group, ***p < .001; unpaired *t*‐test). (C and D) Analysis of MRC complexes assembly by BN‐PAGE in *Oxa1l* cKO mice and littermate control mice (*n* = 3, **p < .01, ***p < .001; unpaired *t*‐test). (E) The relative OXPHOS activities of *Oxa1l* cKO mice and littermate control mice (*n* = 5 mice per group, *p < .05, ***p < .001; unpaired *t*‐test). (F) Oxygen consumption rates of *Oxa1l* cKO mice compared with littermate control mice (*n* = 5 mice per group, *p < .05, ***p < .001; unpaired *t*‐test). (G) Plasma citrate levels in control and *Oxa1l* cKO mice (*n* = 6; unpaired *t*‐test). (H) Pyruvic acid levels in plasma of control and *Oxa1l* cKO mice (*n* ≥ 5; *p < .05; unpaired *t*‐test). (I) The NAD^+^/NADH ratios in gastrocnemius muscles of control and *Oxa1l* cKO mice (*n* ≥ 5; **p < .01; Mann–Whitney *U* test). (J) Lactate concentrations measured in plasma of control and *Oxa1l* cKO mice (*n* = 6; *p < .01; unpaired *t*‐test). (K) Creatine kinase concentrations in control and *Oxa1l* cKO mice's plasma (*n* = 6; *p < .01; Mann–Whitney *U* test). (L) The latency across the balance beam in *Oxa1l* cKO mice and littermate control mice (*n* ≥ 10; *p < .05; Mann–Whitney *U* test). (M) The number of footslips across the balance beam in *Oxa1l* cKO mice and littermate control mice (*n* ≥ 10; *p < .05; unpaired *t*‐test). (N) Grip strength was measured in Oxa1l cKO mice and littermate control mice (*n* ≥ 10; ***p < .001; unpaired *t*‐test). (O) Modified Gomori trichromatic staining in gastrocnemius muscles of control and *Oxa1l* cKO mice. (P) COX‐SDH double staining in gastrocnemius muscles of control and *Oxa1l* cKO mice. (Q) Representative electron micrographs of skeletal muscle sections from control and *Oxa1l* cKO mice. Scale bars, 5 µm (left panel), 2 µm (right panel). All data are presented as mean ± SEM.

To further interrogate the causality of *Oxa1l* variations, skeletal muscle cKO *Oxa1l^f/f,ACAT1‐cre^* mice (Figure S7) was generated. According to western blotting data, the levels of Oxa1l protein in gastrocnemius muscle tissues isolated from *Oxa1l^f/f,ACAT1‐cre^* mice were considerably lower than in age‐matched *Oxa1l^f/f^* mice (Figure 5A,B). Consistent with what we found in OXA1L‐deficient myotubes and KO IHSMC, the steady state levels of multiple subunits encoded by both nDNA (Ndufb8, Uqcrc2, Cox2, Atp5a1) and mtDNA (Nd1, Nd2, Cytb, Atp8) in gastrocnemius muscle tissues of *Oxa1l^f/f,ACAT1‐cre^* mice were significantly decreased. Sdh2 (CII) expression showed no major difference between *Oxa1l^f/f,ACAT1‐cre^* and *Oxa1l^f/f^* mice (Figure S8A–D). Furthermore, the assembly of CI, CIII, CIV and CV are significantly decreased in gastrocnemius muscle tissues of *Oxa1l^f/f,ACAT1‐cre^* mice compared with *Oxa1l^f/f^* mice (Figure 5C,D). Meanwhile, CIV and CV activities in skeletal muscle tissues extracted from *Oxa1l^f/f,ACAT1‐cre^* mice were considerably lower than in *Oxa1l^f/f^* mice (Figure 5E). Consistent with this dysfunction, basal and maximal OCR were markedly diminished in *Oxa1l^f/f,ACAT1‐cre^* mice compared with *Oxa1l^f/f^* mice (Figure 5F), indicating impaired OXPHOS capacity. Notably, while citrate levels, a key tricarboxylic acid cycle metabolite, remained constant between groups (Figure 5G), *Oxa1l* cKO mice displayed elevated pyruvate concentrations (Figure 5H), accompanied by a reduced NAD⁺/NADH ratio (Figure 5I). Serum lactic acid content in *Oxa1l* cKO mice was meaningfully higher than that in control mice, confirming the findings in OXA1L defective cells and mimicking the hyperlactatemia in our patient (Figure 5J). Importantly, serum CK level was raised in *Oxa1l* KO mice, likely reflecting muscular injury (Figure 5K).

To study whether the *Oxa1l* deficit affected motor capabilities in mice, a variety of behavioural assays were carried out in *Oxa1l^f/f,ACAT1‐cre^* and *Oxa1l^f/f^* mice aged at 3–4 months. Although there was no statistical difference in open‐field experiments, total distance was somewhat decreased in *Oxa1l^f/f,ACAT1‐cre^* mice compared with *Oxa1l^f/f^* mice (Figure S8E), implying that *Oxa1l* deficiency may affect locomotor performance of mice to some extent. A subsequent rotarod test revealed no statistically significant differences in fall latency during acceleration between *Oxa1l^f/f,ACAT1‐cre^* and *Oxa1l^f/f^* mice (Figure S8F). Balance beam test revealed that compared with *Oxa1l^f/f^* mice, the latency across the beam was significantly longer (Figure 5L), and the number of slips was also increased (Figure 5M) in *Oxa1l^f/f,ACAT1‐cre^* mice, signifying impaired motor coordination and balancing ability. In grip test, the muscle strength of the *Oxa1l^f/f,ACAT1‐cre^* mice was significantly reduced, compared with *Oxa1l^f/f^* mice (Figure 5N). Together, the above behavioural results indicated that *Oxa1l* deficiency may impair motor coordination function and skeletal muscle strength.

To examine the morphological changes in the gastrocnemius muscle of Oxa1l‐deficient mice, we first stained it with haematoxylin and eosin and discovered no noticeable morphological abnormalities in the gastrocnemius muscle of *Oxa1l^f/f,ACAT1‐cre^* mice compared with *Oxa1l^f/f^* mice (Figure S8G). Modified Gomori trichrome (MGT) staining showed absence of ragged‐red fibres in both groups (Figure 5O). However, COX/SDH double staining demonstrated COX^−^ fibres and reduced COX activity in *Oxa1l^f/f,ACAT1‐cre^* mice versus controls (Figure 5P). Ultrastructural analysis via transmission electron microscopy revealed well organised myofibrils with aligned Z lines in *Oxa1l^f/f^* mice; however, narrow and sparse myofibrils with partially fractured Z‐lines were more commonly observed in *Oxa1l^f/f,ACAT1‐cre^* mice. Moreover, more swollen, cristae ruptured or disappeared, and even vacuolated mitochondria were detected in skeletal muscle of *Oxa1l^f/f,ACAT1‐cre^* mice than in controls (Figure 5Q). These pathological alterations implied that *Oxa1l* deficiency disrupted the ultrastructure of skeletal muscle and mitochondria.

### 3.5 Transcriptomic profiles of patient‐derived myotubes and *OXA1L* KO IHSMC

**FIGURE 6:**
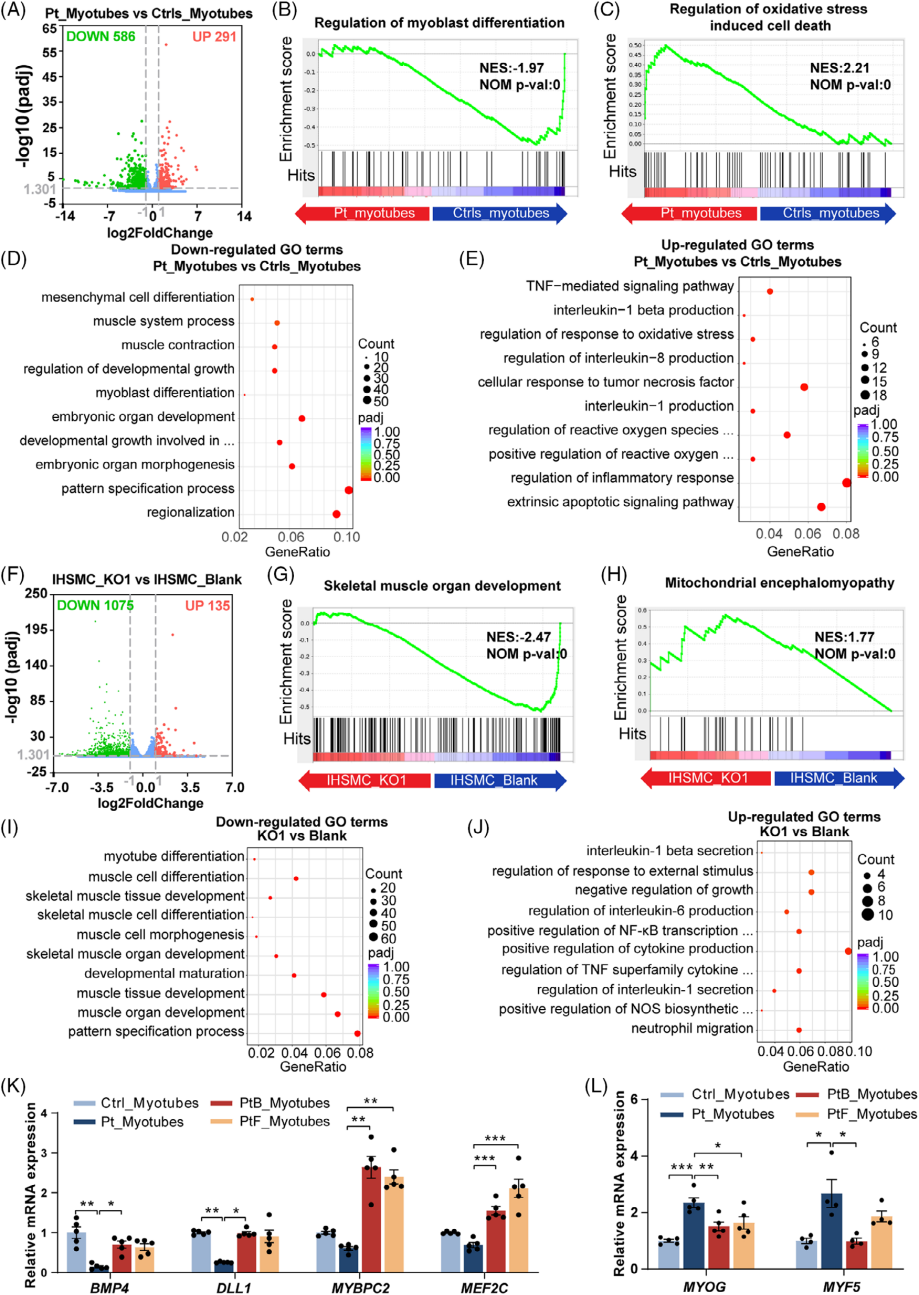
Transcriptomic analysis in patient‐derived myotubes and *OXA1L‐*deficient IHSMC. (A) Volcano plots for changed gene levels in patient‐derived myotubes. (B and C) Enrichment of genes associated with myoblast differentiation (B) and oxidative stress induced cell death (C) in patient‐derived myotubes by gene set enrichment analysis (GSEA). (D and E) Gene ontology (GO) analysis showing significantly down‐regulated (D) and up‐regulated (E) biological processes in patient‐derived myotubes. (F) Volcano plots for altered gene levels in *OXA1L* KO1 IHSMC. (G and H) Enrichment of genes related to skeletal muscle organ development (H) and mitochondrial encephalomyopathy (I) in *OXA1L* depleted IHSMC by GSEA. (I and J) GO analysis displaying meaningfully down‐regulated (J) and up‐regulated (K) biological processes in *OXA1L* depleted IHSMC. (K) qRT‐PCR analysis for the expression of representative down‐regulated myoblast differentiation genes, normalised to β‐actin expression. Data are presented as mean ± SEM (*n* = 5; *p < .05, **p < .01, ***p < .001; Kruskal–Wallis test with Dunn's multiple comparisons test for *BMP4*, *DLL1* and *MYBPC2*, and one‐way ANOVA with Bonferroni post hoc test for *MEF2C*). (L) qRT‐PCR analysis for the expression of *MYF5* and *MYOG*, normalised to β‐actin expression Data are presented as mean ± SEM (*n* ≥ 4; *p < .05, **p < .01, ***p < .001; one‐way ANOVA with Bonferroni post hoc test for *MYOG*, and Kruskal–Wallis test with Dunn's multiple comparisons test for *MYF5*).

To further explore the molecular alterations caused by OXA1L deficiency, we conducted RNA sequencing (RNA‐seq) on patient‐derived myotubes and OXA1L KO IHSMC, respectively. As shown in Figure 6A, there are 586 down‐regulated genes and 291 up‐regulated genes in patient‐derived myotubes (cutoff = 2.0, *p* < .05) versus controls at differentiation day 8. GSEA demonstrated the down‐regulated change of myoblast differentiation genes in patient‐derived myotubes (Figure 6B). While genes that regulate oxidative stress‐induced cell death were up‐regulated in patient‐derived myotubes (Figure 6C). Gene ontology (GO) analysis identified embryonic organ development, myoblast differentiation, muscle contraction and muscle system process as primary targets of transcriptional repression (Figure 6D). Extrinsic apoptotic signalling pathway, and reactive oxygen species (ROS) biosynthetic process were among the most affected biological processes in up‐regulated genes (Figure 6E). Specifically, key myogenic regulators, such as *MYBPC2*, *BMP4*, and *TBX3*, were down‐regulated (Figure S9B), while many ROS and apoptotic regulatory genes, such as *PTX3*, *TNFRSF1B*, *ICAM1*, and *CYP1B1* were up‐regulated in patient‐derived myotubes (Figure S9C).

We also found 1075 down‐regulated genes and 135 up‐regulated genes (cutoff = 2.0, *p* value < .05) in *OXA1L* KO IHSMC (Figure 6F). GSEA revealed down‐regulated changes in skeletal muscle organ development genes (Figure 6G) and up‐regulated changes in mitochondrial encephalomyopathy associated genes (Figure 6H) in *OXA1L* KO IHSMC. Down‐regulated genes, such as *PAX7*, *MEF2C*, and *MYH14* were primarily associated with muscle tissue development and skeletal muscle differentiation (Figures 6I and S9D), and up‐regulated genes, such as *GPX3*, *TNFSF15*, and *CYP1B1* were enriched in biological processes of cytokine modulation and NF‐κB activation (Figures 6J and S9E) in *OXA1L* KO IHSMC. Of note, KEGG analysis also confirmed that up‐regulated genes were predominantly involved in NF‐κB signalling pathway in patient‐derived myotubes (Figure S9A). Furthermore, GO similarity analysis using simplifyEnrichment method revealed that similar down‐regulated biological processes were enriched in development and differentiation (Figure S10A), and similar up‐regulated biological process were enriched in apoptosis in the patient‐myotubes and *OXA1L* KO IHSMC (Figure S10B).

We further verified that genes regulating myoblast differentiation and muscle contraction, such as *BMP4*, *DLL1*, *MYBPC2*, and *MEF2C*, were down‐regulated in patient‐derived myotubes by qPCR (Figure 6K). Furthermore, the expression levels of myoblast regulatory factor MYF5, a hallmark of proliferating myogenic cells, and MYOG, a marker of early differentiation,^27^, ^28^ were higher in patient differentiated myotubes compared with controls (Figure 6L), indicating that altered myogenic programming may present in patient‐derived myotubes.

### 3.6 OXA1L deficiency induced ROS production, NF‐κB activity and apoptosis

**FIGURE 7:**
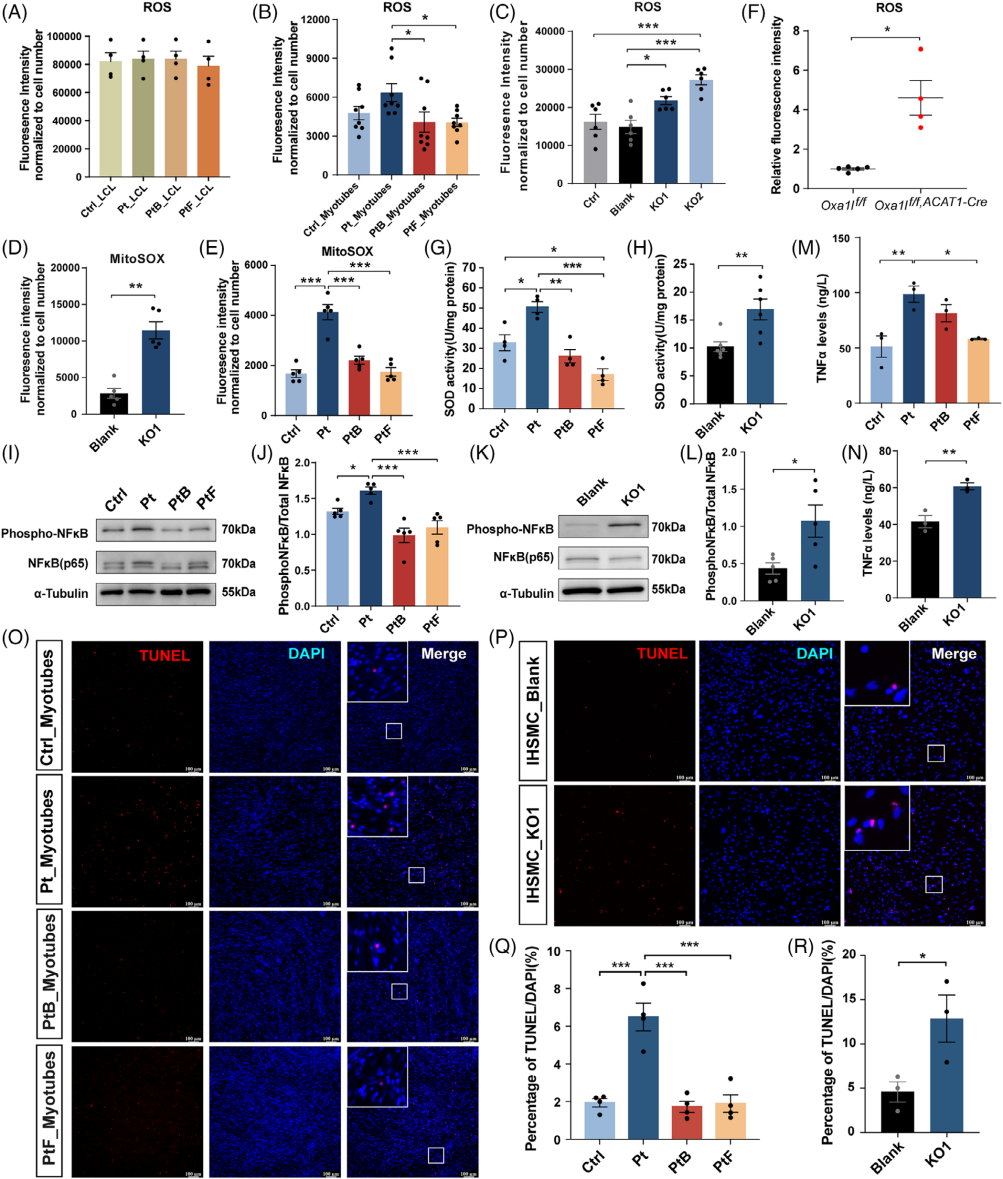
Influences of *OXA1L* deficiency on ROS level, NF‐κB activity and cell apoptosis. (A) Statistics of ROS levels in patient‐derived LCLs (*n* = 3; one‐way ANOVA with Bonferroni post hoc test). (B) Detection of ROS levels in patient‐derived myotubes (*n* = 8; *p < .05; Kruskal–Wallis test with Dunn's multiple comparisons test). (C) Quantification of ROS levels in *OXA1L* depleted IHSMC (*n* = 6; *p < .05, ***p < .001; one‐way ANOVA with Bonferroni post hoc test). (D) MitoSOX fluorescence intensity in *OXA1L*‐KO1 IHSMC (*n* = 5; **p < .01; Mann–Whitney *U* test). (E) MitoSOX fluorescence intensity in patient‐derived myotubes (*n* = 5; ***p < .001; one‐way ANOVA with Bonferroni post hoc test). (F) ROS concentrations measured in skeletal muscle of control and *Oxa1l* cKO mice (*n* ≥ 4; **p < .01; unpaired *t*‐test). (G) Quantification of SOD activity in patient‐derived myotubes (*n* = 4; *p < .05, **p < .01, ***p < .001; one‐way ANOVA with Bonferroni post hoc test). (H) Quantification of SOD activity in *OXA1L* depleted IHSMC (*n* = 6; **p < .01; unpaired *t*‐test). (I and J) Expression of proteins was measured by western blotting using anti‐NF‐κB (p65) and anti phospho‐NF‐κB antibodies in patient‐derived myotubes (*n* = 3; *p < .05, **p < .01; one‐way ANOVA with Bonferroni post hoc test). (K and L) Expression of proteins was measured by western blotting using anti‐NF‐κB (p65) and anti phospho‐NF‐κB antibodies in *OXA1L* depleted IHSMC (*n* = 5; *p < .05; unpaired *t*‐test). (M) TNFα levels in patient‐derived myotubes (*n* = 3; *p < .05, **p < .01; one‐way ANOVA with Bonferroni post hoc test). (N) TNFα levels in *OXA1L*‐KO1 IHSMC (*n* = 3; **p < .01; unpaired *t*‐test). (O–R) Representative images and statistics for TUNEL assay in patient‐derived myotubes (O and Q) and OXA1L depleted IHSMC (P and R). One‐way ANOVA with Bonferroni post hoc test was used for the data presented in Q, and unpaired *t*‐test was used for the data presented in R. All data are shown as mean ± SEM.

The MRC is a known source of intracellular ROS,^29^ and the above RNA‐seq results also suggested ROS biosynthesis pathway activation in OXA1L‐deficient cells. To investigate whether *OXA1L* deficiency led to increased ROS production, we used DCFH‐DA and MitoSOX Red staining. No major difference in intracellular ROS production was observed in patient‐LCLs compared with controls (Figure 7A). In contrast, elevated intracellular and mitochondrial ROS levels were detected in both the patient myotubes and *OXA1L* KO IHSMC, compared with respective controls (Figure 7B–E). Meanwhile, ROS content in *Oxa1l* KO mice gastrocnemius tissues were also enhanced (Figure 7F). Surprisingly, the activity of SOD, an important intracellular antioxidant, was elevated in both the patient myotubes and *OXA1L* KO IHSMC, presumably in a compensatory way (Figure 7G,H).

Established mechanisms indicate ROS‐mediated NF‐κB regulation,^30^, ^31^, ^32^ with concurrent transcriptomic profiling revealing NF‐κB pathway activation in OXA1L‐deficient cells. Western blotting validation demonstrated NF‐κB hyperactivation, evidenced by increased phosphatase NF‐κB (pNF‐κB)/total NF‐κB ratios, suggesting elevated NF‐κB activity in both the patient myotubes (Figure 7I,J) and *OXA1L* KO IHSMC (Figure 7K,L). Furthermore, TNFα levels were markedly elevated in both patient‐derived myotubes (Figure 7M) and *OXA1L* KO IHSMC (Figure 7N) compared with respective controls.

Subsequent TUNEL assays demonstrated significantly increased ratio of TUNEL and DAPI double positive (TUNEL^+^/DAPI^+^) cells in both patient‐derived myotubes (Figure 7O,Q) and *OXA1L* KO IHSMC (Figure 7P,R), compared with that in corresponding controls. In summary, these results suggest that *OXA1L* deficiency may promote ROS production, enhance the activity NF‐κB signalling pathway, and induce cell apoptosis.

### 3.7 Mito‐TEMPO treatment attenuated apoptosis in patient‐derived myotubes and *Oxa1l* cKO mice

**FIGURE 8:**
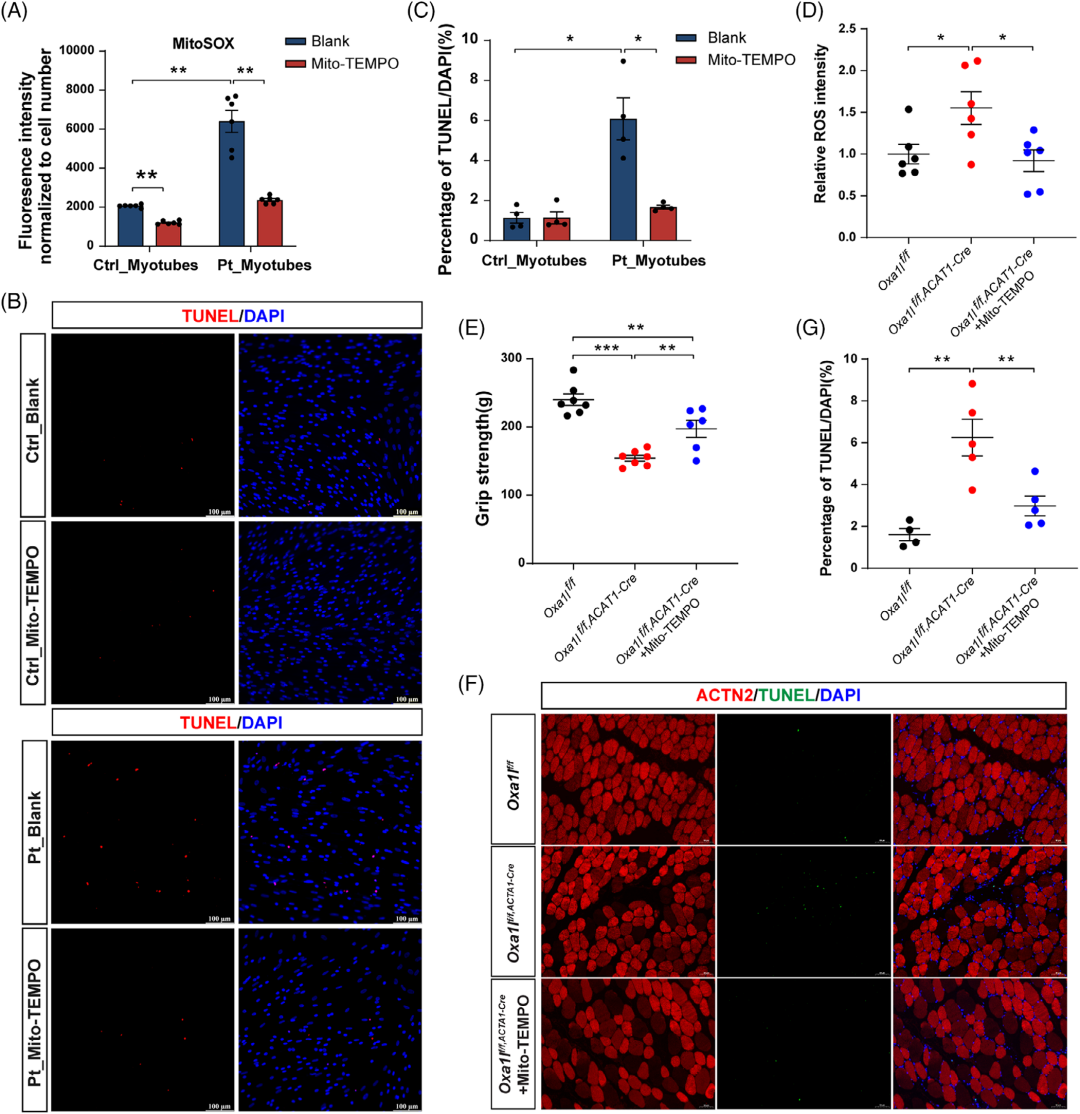
Rescue effects of the antioxidant Mito‐TEMPO in patient‐derived myotubes and *Oxa1l* cKO mice. (A) Statistics of mt‐ROS levels in patient‐derived myotubes with and without Mito‐TEMPO treatment (*n* = 6; **p < .01; Mann–Whitney *U* test). (B and C) Representative images (B) and statistics (C) for TUNEL assay in patient‐derived myotubes with and without Mito‐TEMPO treatment (*n* = 4; *p < .05; Mann–Whitney *U* test). (D) Statistics of ROS levels in gastrocnemius muscles of *Oxa1l* cKO mice with and without Mito‐TEMPO treatment (*n* = 6; *p < .05; one‐way ANOVA with Bonferroni post hoc test). (E and F) Representative images (E) and statistics (F) for TUNEL staining in gastrocnemius muscles of *Oxa1l* cKO mice with and without Mito‐TEMPO treatment (*n* ≥ 4; **p < .01; one‐way ANOVA with Bonferroni post hoc test).

To establish ROS as a causal driver of pathology, patient‐derived myotubes were treated with Mito‐TEMPO (a mitochondria‐targeted antioxidant) during both MPC stages and differentiation. This intervention effectively suppressed mitochondrial ROS accumulation (Figure 8A) and considerably reduced the proportion of TUNEL^+^/DAPI^+^ apoptotic cells in treated cultures (Figure 8B,C). Complementary in vivo studies demonstrated that systemic Mito‐TEMPO administration in *Oxa1l*‐cKO mice reduced ROS levels in the gastrocnemius (Figure 8D), ameliorated grip strength deficits (Figure 8E) and attenuated gastrocnemius muscle apoptosis (Figure 8F,G). Collectively, these findings directly link mitochondrial ROS inhibition to phenotypic amelioration in both cellular and murine models.

## 4 DISCUSSION

In this study, we report the second mitochondrial myopathy case carrying bi‐allelic variants in *OXA1L* gene, one of which is a novel variant. To explore the pathogenicity and potential pathogenesis of *OXA1L* variants, we performed a series of functional experiments using patient‐derived myotubes, *OXA1L* KO IHSMC and cKO mouse model. Our work not only adds to our current knowledge on mitochondrial myopathy associated with bi‐allelic pathogenic variants in *OXA1L*, but also provides a potential target for the intervention of mitochondrial diseases.

Cytochrome *c* oxidase assembly factor 18 (*COX18*, MIM*610428), an insertase critical for cytochrome *c* oxidase assembly, belongs to the Oxa1/Alb3/YidC insertase family and shares partial structural similarity with OXA1L. Notably, bi‐allelic *COX18* pathogenic variants have been molecularly linked to severe mitochondrial disorders characterised by progressive encephalomyopathy and neuropathy, as demonstrated in recent clinical case reports with biochemical validation of respiratory chain deficiencies.^33^, ^34^, ^35^ To our best knowledge, only one family has been previously reported to harbour bi‐allelic variants c.500_507dup, p.(Ser170Glnfs*18) and c.620G>T, p.(Cys207Phe) in the *OXA1L* gene associated with mitochondrial disease.^21^ In line with previous report, our patient harboured the same splicing affected variant c.620G>T, p.(Cys207Phe) as the first patient, as well as a novel frameshift variant c.1163_1164del, p.(Val388Alafs*15), expanding the spectrum of *OXA1L* gene variants. Despite the fact that our findings enhance the association between *OXA1L* variants and mitochondrial diseases, the limited number of reported cases currently precludes definitive genotype–phenotype correlations. According to ACMG/AMP guidelines, both the c.620G>T and c.1163_1164del variants are classified as variants of uncertain significance due to insufficient evidence supporting their pathogenicity in humans. Although in silico and functional evidence suggest these variants disrupt OXA1L function, further validation in expanded patient cohorts is essential to establish a causal link between *OXA1L* variants and mitochondrial disease.

Patient‐derived LCLs exhibited stable MRC subunits expression, while mRNA expressions of some MRC associated genes were reduced. OXA1L defects are likely to disrupt mitochondrial function at both the translational and post‐translational levels, resulting in impaired assembly and stability of MRC. The down‐regulation of mRNA for certain MRC subunits in *OXA1L*‐KO LCLs could be explained by the fact that impaired MRC function may trigger an intracellular feedback mechanism that reduces the accumulation of aberrant proteins by either down‐regulating transcription of the relevant genes or promoting mRNA degradation.

Given the tissue‐specific vulnerability in mitochondrial pathologies, LCLs may not accurately reflect the disease's features. Because of the scarcity of human tissue, defining disease mechanisms in neurons or skeletal muscles remains difficult. Recently, hiPSC derived from individuals with mitochondrial disorders can be differentiated to neurons,^36^ skeletal muscle cells^27^ and cardiomyocytes,^37^ showing tremendous promise in unravelling the pathogenesis of mitochondrial disease. We employed hiPSC‐derived myotube differentiation model to shed light on the morphofunctional pathways underlying mitochondrial myopathy resulting from *OXA1L* bi‐allelic variants. Our results showed severely reduced levels of OXA1L and combined MRC complex defects, as well as lower CIII, CIV and CV enzyme activities in both patient‐derived myotubes and *OXA1L* KO IHSMC. Slightly different with what we reported in *OXA1L* KO IHSMC, the assembly level of CIV in patient‐derived myotubes did not show a significant decrease. Previous research has found that OXA1L deficiency cause tissue‐ and cell‐specific OXPHOS impairment.^20^, ^21^ Thompson et al.^21^ recently discovered decreased complex I/IV/V subunits, along with lower CIV enzyme activity in skeletal muscle of patient harbouring *OXA1L* bi‐allelic variants, whereas a seemingly isolated CI deficiency in brain tissue. Crucially, results on complex abnormalities due to OXA1L deficiency differed amongst laboratories, even within the same cell line. In HEK293 cells with shRNA‐mediated OXA1L knockdown, Stiburek et al.^20^ demonstrated evident abnormalities in CI and CV, although CIV remained unaffected. Thompson et al.,^21^ on the other hand, found significant reductions in complex I, IV and V in OXA1L depleted HEK293 cells. Taken together, several results based on OXA1L variant patients or OXA1L‐deficient cell lines revealed that OXA1L deficiency does lead to MRC abnormalities. The specific pathogenic roles, however, seem to vary by tissue and cell type.

Rescue experiments conducted in *OXA1L* KO IHSMC revealed that the Cys207Phe missense variant, resulting from the c.620C>T variant carried by the patient, partially rescued the OXA1L and complex defects. However, both splicing variant Cys207_Glu254del and frameshift variant Val388Alafs*15 failed to do so. We hypothesise that the observed heterogeneity between *OXA1L* KO IHSMC and patient‐derived myotubes may be attributed to the partial retention of OXA1L function by the Cys207Phe missense variant carried by the patient, which compensates for some of the complex defects. As demonstrated by Thompson et al.,^21^ differential OXA1L depletion levels can indeed lead to distinct biochemical outcomes.

The patient previously reported presenting with severe encephalopathy, muscle weakness, hypotonia, developmental delay and generalised seizures before dying at 5 years.^21^ In contrast, our patient showed motor regression, muscle weakness, hypotonia, lower‐extremity muscle atrophy, but no signs of neurodevelopmental delay have been detected to date. The phenotypic divergence between our patient and the previously reported case likely arises from allele‐ and tissue‐specific thresholds of residual OXA1L function. Tissue‐specific splicing factors may influence alternative splicing outcomes of c.620G>T variant in brain and skeletal muscle, preferring depleting functional OXA1L in skeletal muscle or making brain retain higher levels of the functional p.Cys207Phe isoform. OXA1L displays ubiquitous expression with tissue‐specific abundance, showing the lowest levels in brain tissue and significantly higher expression in skeletal muscle.^21^ This differential expression pattern may render skeletal muscle particularly vulnerable to partial loss‐of‐function *OXA1L* variants, whereas brain tissue maintains preserved OXPHOS capacity even under OXA1L reduction, potentially sustained through compensatory mitochondrial biogenesis. Furthermore, in contrast to the prior case's c.500_507dup frameshift variant, our patient's c.1163_1164del frameshift variant may be more prone to evade nonsense‐mediated decay, allowing residual truncated OXA1L to maintain minimal function while sparing the brain. Given the genotype–phenotype heterogeneity of mitochondrial disease, and the fact that only two cases with *OXA1L* bi‐allelic variants have been reported, determining why our patient has a different phenotype from the first family is tough. Further validation based on additional patient samples, as well as functional investigations to examine the molecular mechanisms behind this variability, are required.

Mouse models are invaluable tools for understanding mitochondrial dysfunction and essential for evaluation of potential intervention strategies.^38^, ^39^, ^40^ To our knowledge, no investigations on Oxa1l defective mice models have been conducted. To clarify the causality and elucidate the underlying pathomechanistic for mitochondrial myopathy caused by *OXA1L* variants, skeletal muscle cKO *Oxa1l^f/f,ACAT1‐cre^* mice was successfully generated. Our findings showed that *Oxa1l* KO mice had significantly reduced steady‐state levels (Figure S6) and assembly (Figure 5C,D) of CI, CIII, CIV and CV, as well as considerably lower enzymatic activities of CIV and CV (Figure 5E), indicating that Oxa1l deficiency indeed affect OXPHOS despite species heterogeneity with human cells. Furthermore, our behavioural findings revealed that *Oxa1l^f/f,ACAT1‐cre^* mice have deficits in motor coordination function and skeletal muscle strength, consisting with our patient's muscle weakness. Serum lactic acid levels were likewise elevated in *Oxa1l^f/f,ACAT1‐cre^* mice, which is consistent with Hyperlactatemia in our patient. In addition, reduced COX activity and disturbed ultrastructures of skeletal muscle and mitochondria were identified in *Oxa1l^f/f,ACAT1‐cre^* mice. The above results revealed that *Oxa1l^f/f,ACAT1‐cre^* mice had a phenotype comparable to patients with mitochondrial myopathy, establishing the association between OXA1L deficiency and mitochondrial disease.

The mechanistic link between OXA1L deficiency‐induced mitochondrial abnormalities and skeletal myopathy remains incompletely understood. Apoptosis, a hallmark of mitochondrial myopathies, is mechanistically driven by bioenergetic collapse, calcium dysregulation and ROS overproduction.^41^ Clinical correlations reveal elevated apoptosis in muscle biopsies from patients with high mtDNA mutation burdens.^42^ Transcriptomic profiling of OXA1L‐deficient cells revealed coordinated up‐regulation of ROS biosynthesis and apoptotic pathways paralleled down‐regulation of skeletal myogenesis‐related genes. This aligns with prior study employing a mitochondrial disease patient‐derived hiPSC‐to‐skeletal muscle differentiation model, which reported delayed myotube formation and aberrant myogenic marker expression.^27^ Furthermore, higher levels of ROS and cell apoptosis, as well as aberrant myogenic marker expression were demonstrated in OXA1L defective myotubes. Rescue experiments further revealed that inhibiting ROS significantly reduced apoptosis in both patient‐derived myotubes and in *Oxa1l* cKO mice. The ROS paradox in muscle biology‐essential for homeostasis yet destructive in excess‐manifests through multiple pathological axes: impaired myoblast differentiation, apoptosis induction, autophagic dysregulation, inflammatory activation and calcium mishandling.^43^, ^44^, ^45^ Studies have indicated that high concentrations of ROS can cause impaired myoblast function and increased myoblast death.^46^ Myoblasts lacking the mitochondrial antioxidant glutathione peroxidase (GPx) exhibited impaired proliferation, differentiation and myotube formation, as well as increased cell apoptosis.^47^ Transcriptomic and protein analyses in OXA1L‐deficient cells further identified hyperactivated NF‐κB signalling, an essential redox‐sensitive transcription factor,^31^, ^43^, ^48^ known to impair myotube differentiation by suppressing MyoD expression^49^, ^50^, ^51^ and activating YY1 expression.^52^ Notably, blocking the NF‐κB signalling pathway prevented oxidative stress‐induced apoptosis in myoblasts via reducing Caspase‐3/9 activation.^30^ Based on our findings, we speculate that NF‐κB signalling pathway activation mediated by elevated ROS from OXA1L deficiency‐induced OXPHOS impairment may influence myogenic programming and apoptosis, which may be a potential pathogenic mechanism leading to mitochondrial myopathy. Existing literature highlights mitochondria as critical regulators of myoblast differentiation through bioenergetic supply and redox signalling.^53^, ^54^, ^55^ While OXA1L directly orchestrates respiratory chain assembly and OXPHOS efficiency, rendering its deficiency a plausible disruptor of skeletal myogenic programming, our current evidence remains transcriptomically inferred, necessitating functional validation of proposed mechanisms. Given the functional diversity of OXPHOS, further research is warranted to clarify other undiscovered molecules or signalling pathways that may be implicated in the initiation and progression of OXA1L‐related mitochondrial myopathy.

## 5 CONCLUSION

In summary, we identified bi‐allelic *OXA1L* variants in a Chinese girl with mitochondrial myopathy, which broadens the mutation spectrum and reinforces the genotype–phenotype association between *OXA1L* variations and mitochondrial diseases. Our functional study highlights that the dysregulation of NF‐κB signalling pathway triggered by excessive ROS resulting from OXPHOS defects may be the underlying pathogenic mechanism of mitochondrial myopathy caused by *OXA1L* variants, shedding new lights on understanding these processes and developing new therapies.

## AUTHOR CONTRIBUTIONS

Y.Z.: conceptualisation, data curation, funding acquisition, methodology, writing—original draft, writing—review and editing. Q.W.: formal analysis, investigation and methodology. Y.W.: investigation and methodology. Y.F.: formal analysis and resources. D.Y.: investigation and methodology. X.L.: methodology. Y.C.: investigation. T.H.: investigation and methodology. N.L.: investigation and methodology. W.D.: investigation and methodology. H.F.: conceptualisation, methodology, writing—review and editing. Y.Y.: conceptualisation, funding acquisition, resources, supervision and writing—review and editing.

## CONFLICT OF INTEREST STATEMENT

The authors declare no conflicts of interest.

## ETHICS STATEMENT AND CONSENT TO PARTICIPATE

Written informed consent for the patient involvement in the research and publication was obtained from the parents. The study was carried out in accordance with the Helsinki Declaration principles and was approved by the Ethics Committee of Xinhua Hospital, School of Medicine, Shanghai Jiao Tong University (No. XHEC‐D‐2024‐109).

## CONSENT FOR PUBLICATION

Data/manuscript publication was approved by all authors.

## Supporting information



Supporting Information

## Data Availability

Data presented in this study are available from the corresponding author on reasonable request.
