# Supplementary material for: OXA1L deficiency causes mitochondrial myopathy via reactive oxygen species regulated nuclear factor kappa B signalling pathway
Source: Clin Transl Med. 2025 Jun 23;15(6):e70385. doi: 10.1002/ctm2.70385 (PMC12185910; doi:10.1002/ctm2.70385)
Supplement: Supplementary file 1 — Supporting Information [file CTM2-15-e70385-s001.docx]

**Supplemental information**

**
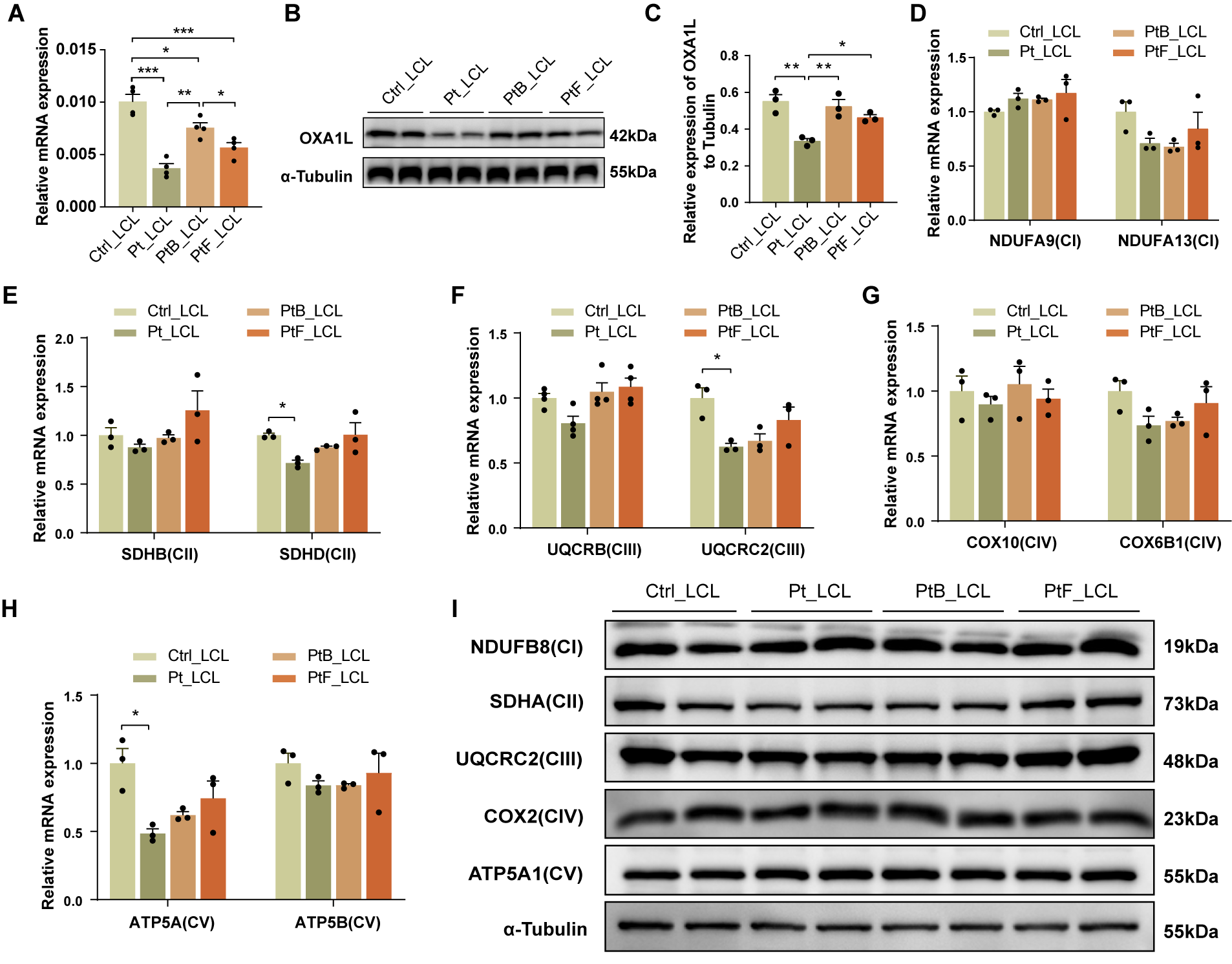
**

**Figure S1. Reduced expression of OXA1L and complexes in patient specific LCL. (A)** qRT-PCR analysis of *OXA1L* mRNA expression in patient (Pt) and controls (Ctrl, PtF, PtB) derived LCL, normalized to β-actin (n = 3 replicates; *P<0.05, **P<0.01, ***P<0.001; one-way ANOVA with Bonferroni post hoc test). **(B, C)** The expression of OXA1L protein in patient (Pt) and controls (Ctrl, PtF, PtB) derived LCL, normalized to α-Tubulin (n = 3 replicates; *P<0.05, **P<0.01; one-way ANOVA with Bonferroni post hoc test). **(D-H)** qRT-PCR assessment of MRC complex subunits-associated genes in patient (Pt) and controls (Ctrl, PtF, PtB) derived LCL (n = 3 replicates; *P<0.05; one-way ANOVA with Bonferroni post hoc test for SDHB, UQCRC2, COX10, COX6B1, and ATP5A1, and Kruskal-Wallis test with Dunn’s multiple comparisons test for NDUFA9, NDUFA13, SDHD, UQCRB, and ATP5B). **(I)** Expression of MRC complex subunits was measured by western blotting. All data are shown as mean ± SEM.

**
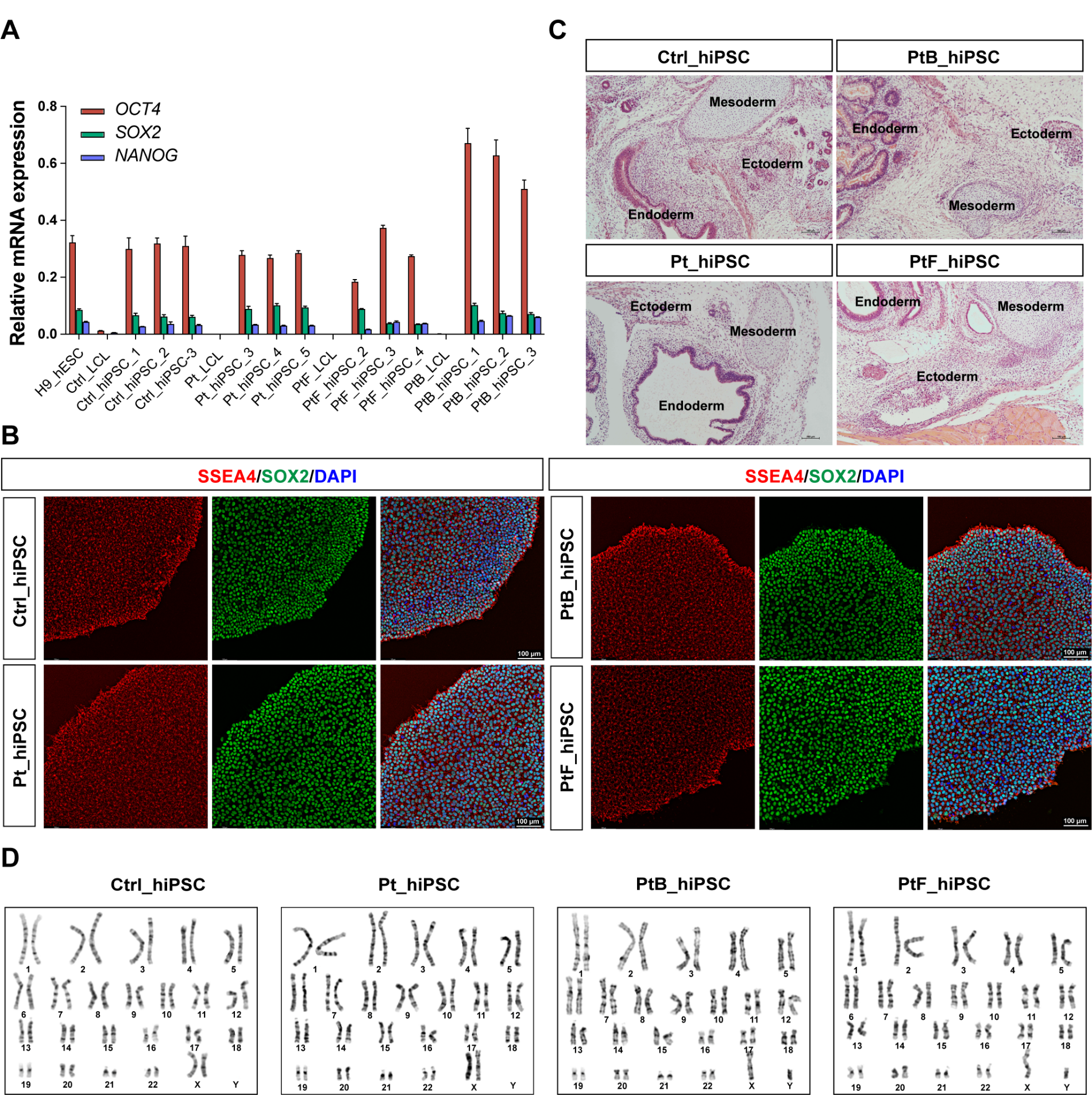
**

**Figure S2. Pluripotency of patient-specific hiPSCs. (A)** qRT-PCR analysis for the expression of pluripotency markers, normalized to β-actin expression. Data are presented as mean ± SEM from three independent experiments. **(B)** Immunostaining of pluripotency markers such as SSEA4 (red) and SOX2 (green). Scale bar, 100μm. **(C)** Images of teratoma with tissues signifying all three germ layers (ectoderm, mesoderm, and endoderm). Scale bars, 100 μm. **(D)** Karyotyping analysis of hiPSCs after 10 passages.

**
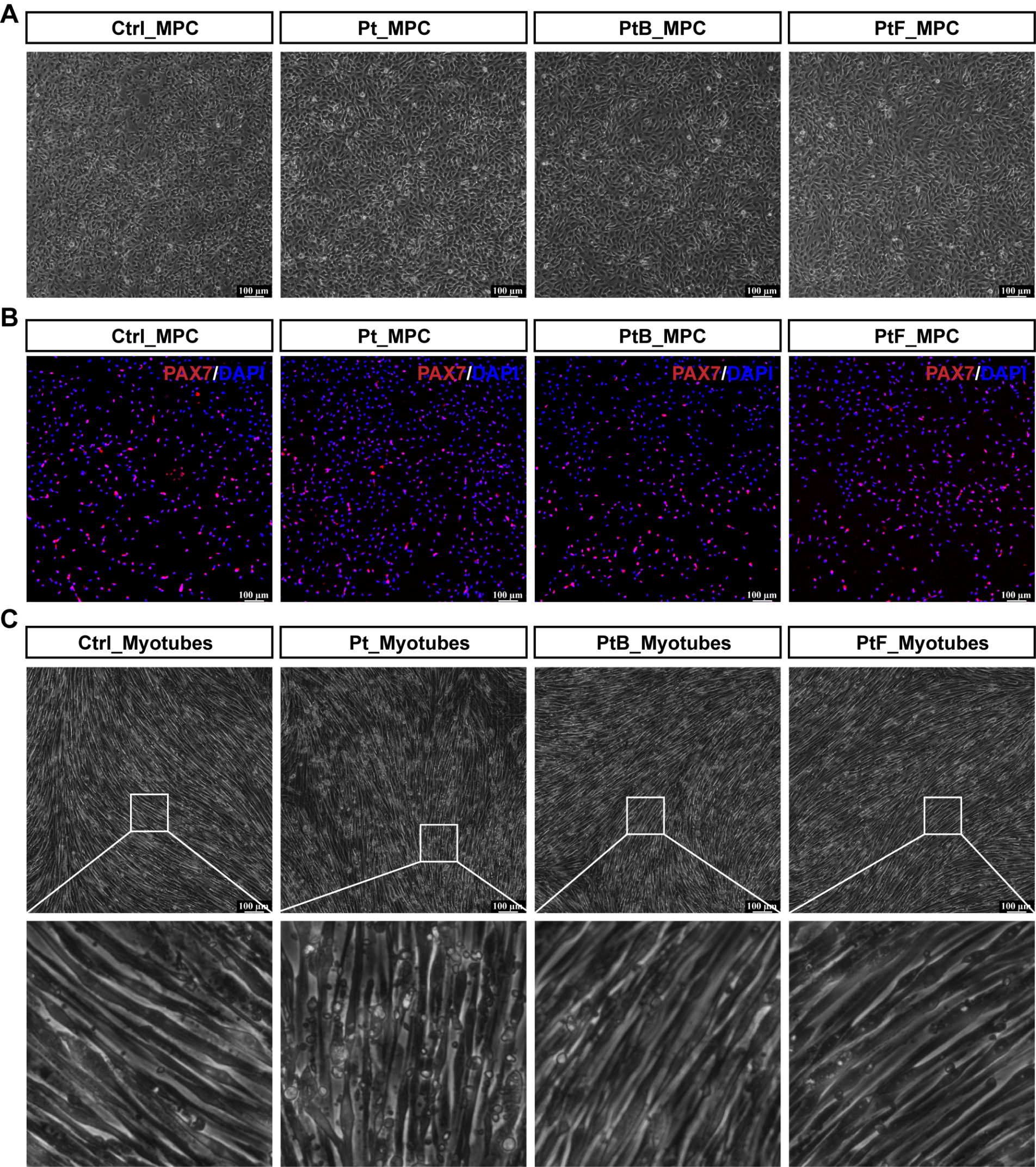
**

**Figure S3. Morphology of patient derived myogenic progenitor cell and myotubes. (A)** Representative images showing passage 3 myogenic progenitor cells (MPCs) differentiated from hiPSCs. Scale bars, 100 μm. **(B)** Immunostaining of myogenic marker PAX7 (red) in MPCs. Scale bar, 100μm. **(C)** Representative images showing myotubes differentiated from hiPSCs derived MPCs on day 8. Scale bars, 100 μm.

**
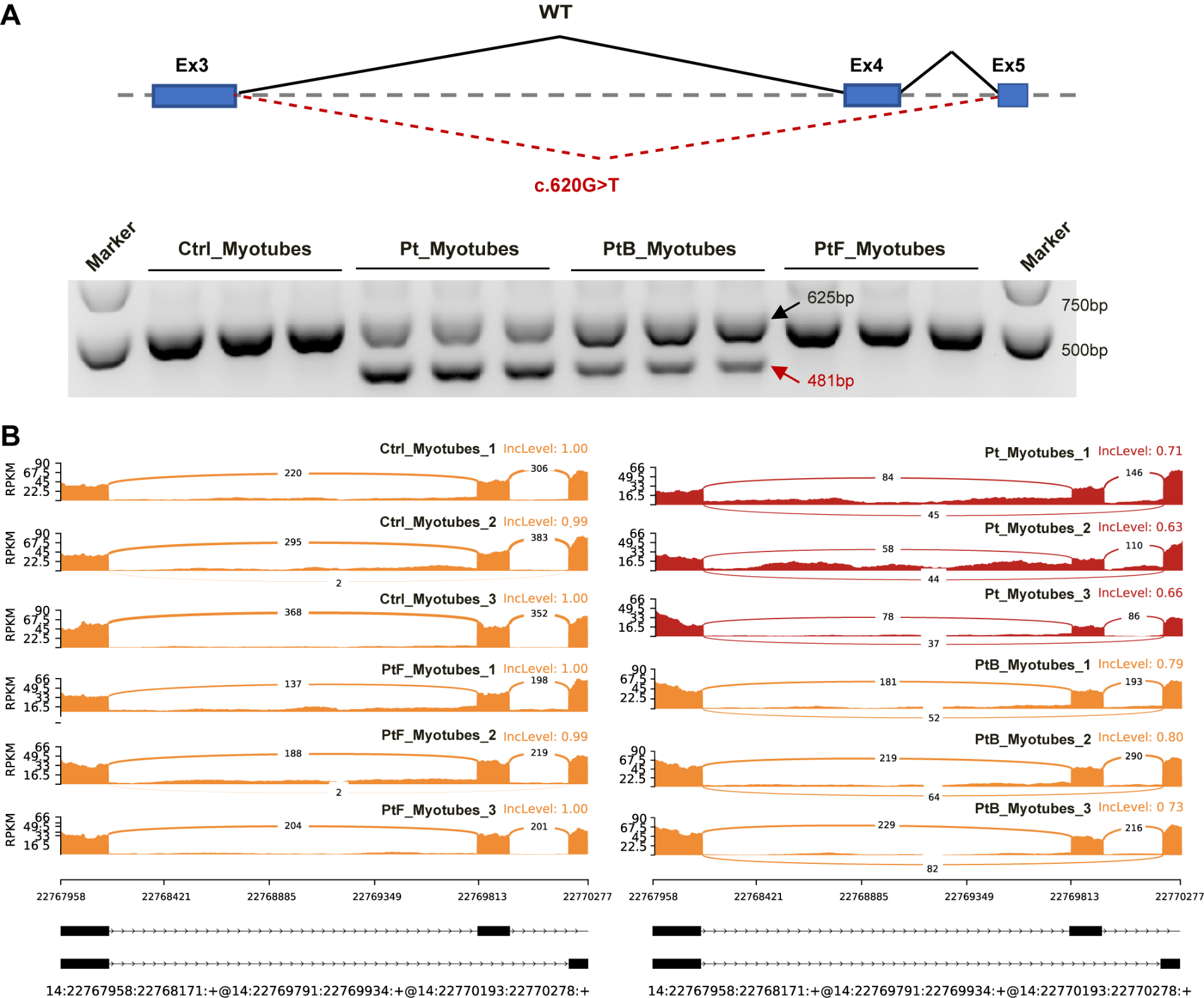
**

**Figure S4. Altered *OXA1L* gene splicing patterns in myotubes harboring the c.620G>T variant. (A)** Agarose gel electrophoresis examination of alterations in the *OXA1L* gene’s splicing patterns in patient derived myotubes. **(B)** RNA-Seq reads and predicted exon-skipping reads of *OXA1L* exon 4 in controls and Pt derived myotubes as determined by rMATS analysis.

**
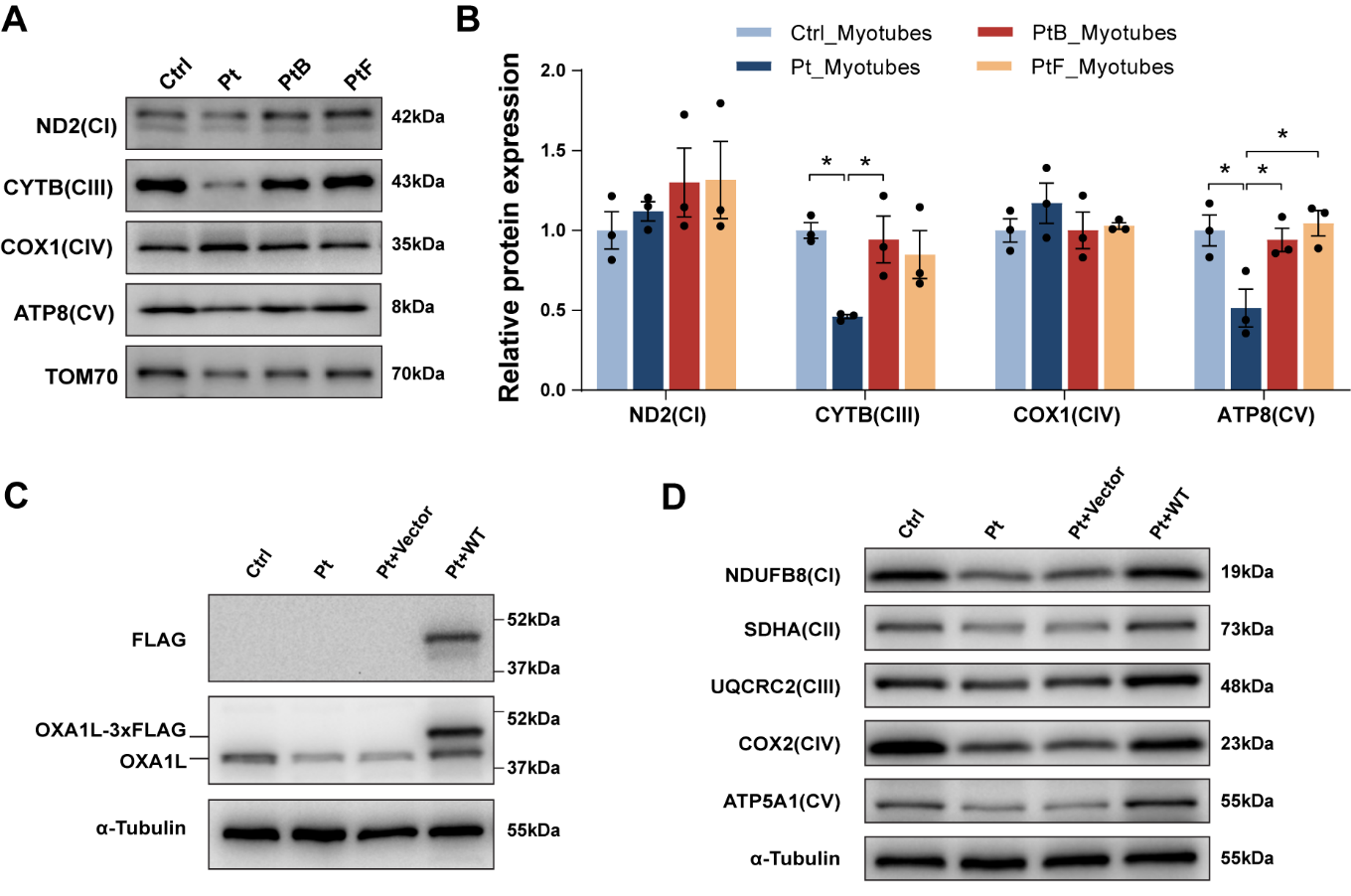
**

**Figure S5.** **Analysis of steady-state levels of the mtDNA-encoded subunitsin patient myotubes and** **evaluation of rescue effects by overexpressing wild-type OXA1L.** Representative images **(A)** and statistics **(B)** of mt-DNA encoded MRC subunits measured by western blotting analysis in myotubes (Data are presented as mean ± SEM. n=3; *P<0.05; one-way ANOVA with Bonferroni post hoc test for ND2, CYTB, and ATP8, and Kruskal-Wallis test with Dunn’s multiple comparisons test for COX1). **(C)** The expression of internal OXA1L and external FLAG in Pt-derived myotubes transfected with FLAG-tagged OXA1L WT plasmids. **(D)** The expression of MRC subunits detected by western blotting in Pt-derived myotubes transfected with WT plasmids.

**
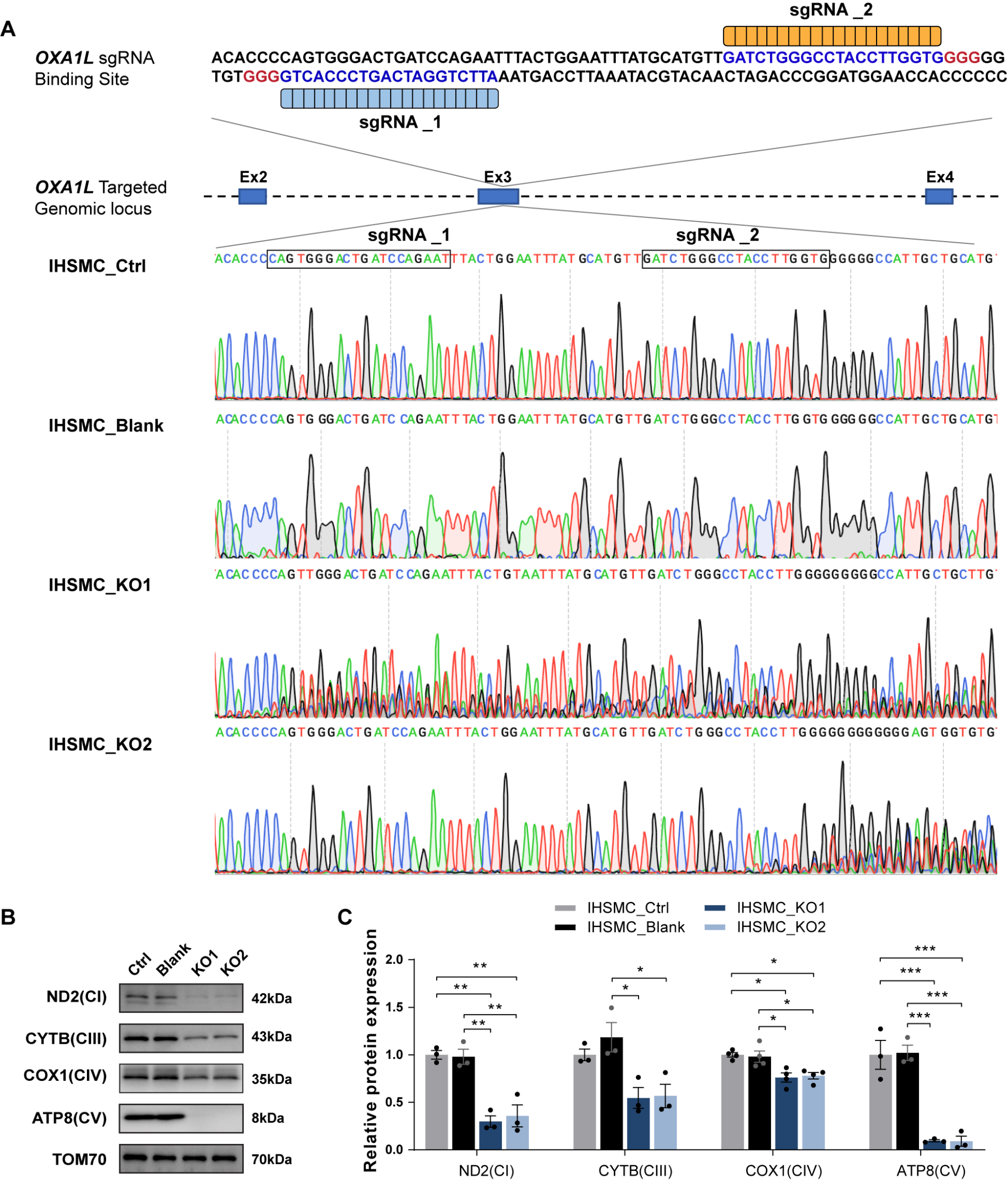
**

**Figure S6.Generation of OXA1L knockout IHSMC using CRISPR/Cas9 strategy and analysis of steady-state levels of the mtDNA-encoded subunits in IHSMC. (A)** Indel mutations near sgRNA1 or sgRNA2 sequences were verified by Sanger sequencing. **(B, C)** Representative images (B) and analysis (C) of mt-DNA encoded MRC subunits measured by western blotting in IHSMC. Data are presented as mean ± SEM. n=3; *P<0.05, **P<0.01, ***P<0.001, one-way ANOVA with Bonferroni post hoc test.

**
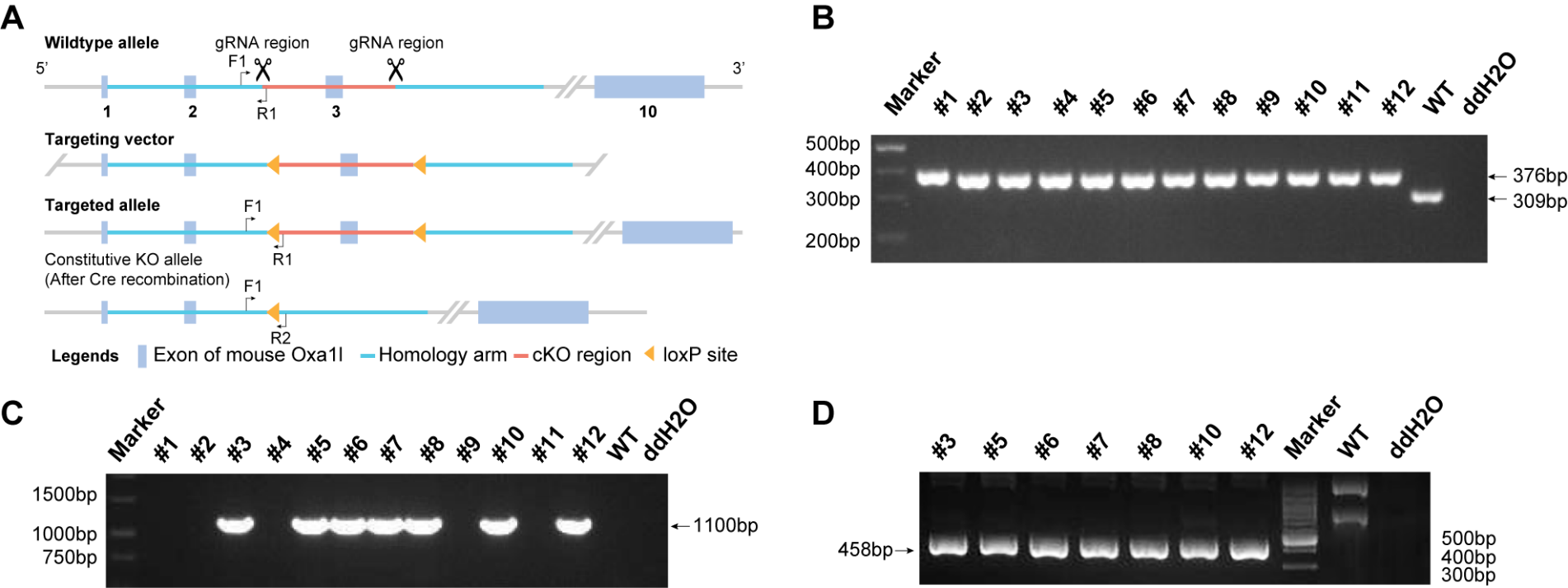
**

**Figure S7. Construction strategy and genotyping of the *Oxa1l* conditional knockout mouse model using CRISPR/Cas9-mediated genome engineering. (A)** A schematic diagram of the construction strategy for the *Oxa1l* conditional knockout mouse model. **(B)** Identification of loxp insertions using agarose gel electrophoresis. **(C)** The presence of the ACTA1-CreEsr1 transgene was identified by agarose gel electrophoresis. **(D)** Gel electrophoresis identification of a 458 bp product resulting from exon 3 excision in the *Oxa1l* gene.

**
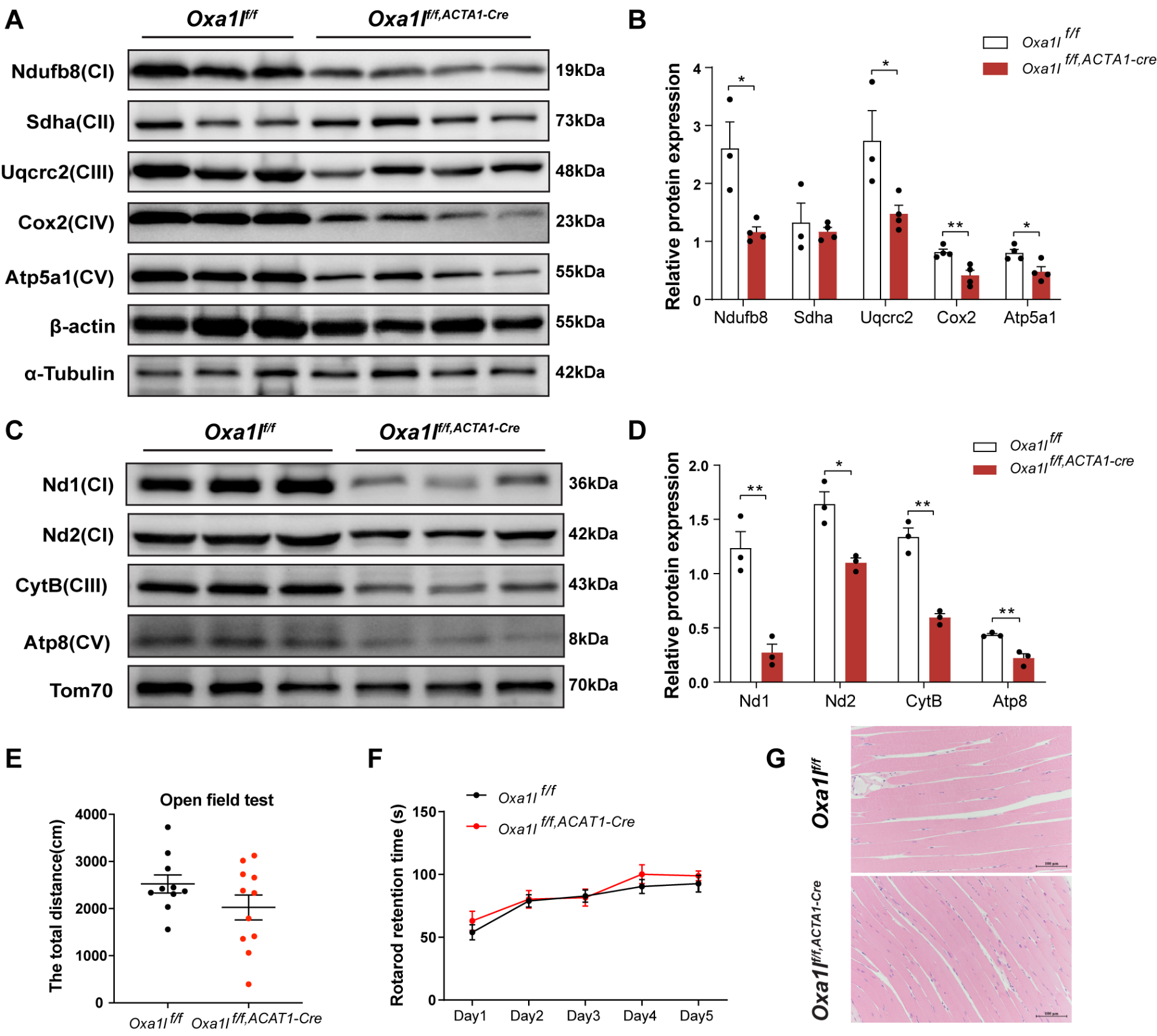
**

**Figure S8. Analysis of steady-state levels of MRC complexes, behavioral assessment, and H&E staining in *Oxa1l* cKO mice. (A-D)** Representative images (A, C) and statistics (B, D) of MRC complex subunits analyzed by western blotting in *Oxa1l* cKO mice and littermate control mice. Data are presented as mean ± SEM (n ≥ 3 mice per group; *P<0.05, **P<0.01; unpaired t-test). (**E**)The total distance of *Oxa1l* cKO mice (n=11) and littermate control mice (n=10) moved in OFT. (**F**) The retention times of falling from an accelerating rotarod in *Oxa1l* cKO mice (n=11) and littermate control mice (n=10). (**G**) H&E staining in gastrocnemius muscles of control and *Oxa1l* cKO mice.

**
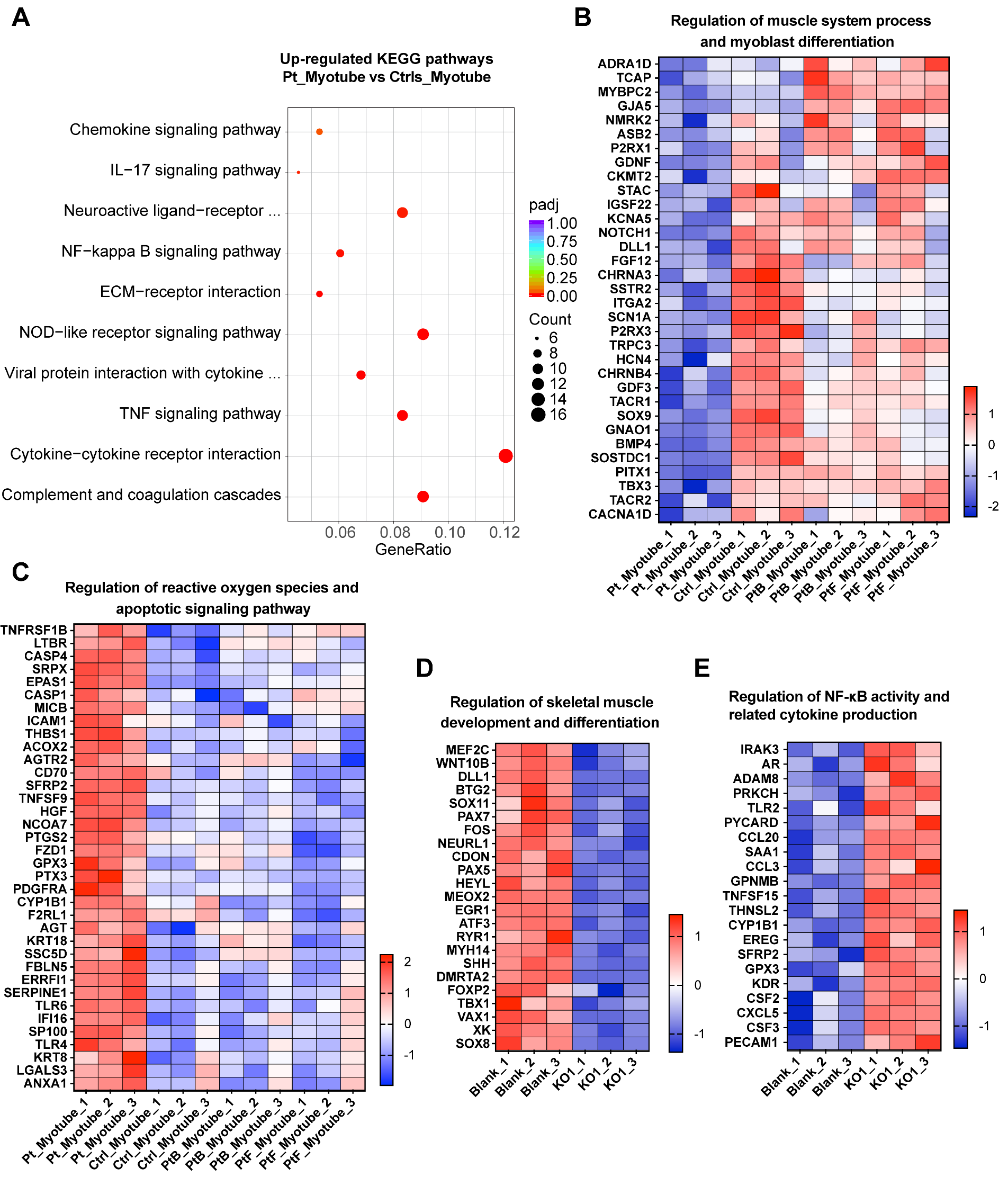
**

**Figure S9. Analysis for changed genes in OXA1L deficient cells.** **(A)** KEGG pathway enrichment for upregulated genes in patient derived myotubes. **(B)** Heatmap for changed genes involved in myoblast differentiation in patient derived myotubes. **(C)** Heatmap for changed genes associated with reactive oxygen stress in patient derived myotubes. **(D)** Heatmap for down-regulated genes in *OXA1L* knockout IHSMC. **(E)** Heatmap for up-regulated genes in *OXA1L* knockout IHSMC.

**
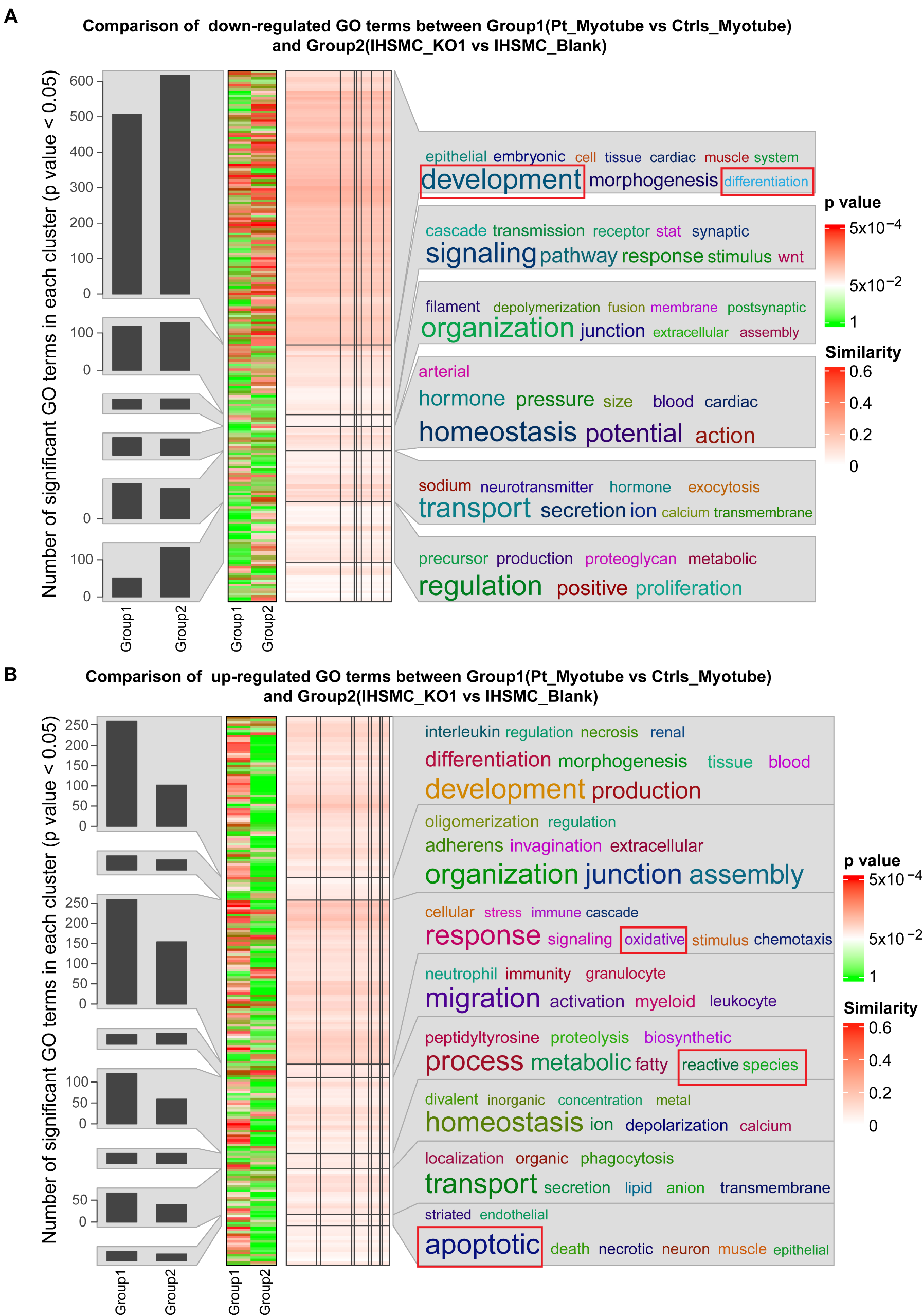
**

**Figure S10. GO term similarity analysis on *OXA1L* deficient myotubes and IHSMC. (A, B)** Analysis on GO term similarity showed significant enrichment in development (A) and apoptosis (B) between Group 1 and Group 2.

**Table S1. *OXA1L* variant sequencing primers**

| **Primer** | **Forward** | **Reverse** |
| --- | --- | --- |
| OXA1L-620-Variant | CATTCCCCTGACCTTATTTCTG | TGTGGCAACTTCTGATTTACATTGT |
| OXA1L-1163_1164-Variant | GATGCCCCTGATAACCTTGC | AGACCCCTTTCCCTAGTCCTTT |

**Table S2. Primers for** **alternative splicing analysis**

| **Primer** | **Forward** | **Reverse** |
| --- | --- | --- |
| OXA1L-620-AS | AAGTCCAGGTTCAGGCCCCT | GCACCTAGCTCAAGAACAGCCCA |

**Table S3. Targeted gRNA sequences for the generation of knockout IHSMC**

| **gRNA** | **sequence** |
| --- | --- |
| sgRNA-1 | ATTCTGGATCAGTCCCACTGGGG |
| sgRNA-2 | GATCTGGGCCTACCTTGGTGGGG |

**Table S4. Quantitative real-time PCR Primers**

| **Primer** | **Forward** | **Reverse** |
| --- | --- | --- |
| *β-Actin* | GGACTTCGAGCAAGAGATGG | AGCACTGTGTTGGCGTACAG |
| *OCT4* | GCAAAGCAGAAACCCTCGTG | CACACTCGGACCACATCCTT |
| *SOX2* | ATGGACAGTTACGCGCACAT | CGAGCTGGTCATGGAGTTGT |
| *NANOG* | CTTCACCTATGCCTGTGATTTG | GCTGAGGTATTTCTGTCTCTG |
| *OXA1L* | TTGCAGAAGTCCAGGTTCAGG | GTCCCACTGGGGTGTATGAC |
| *NDUFA9* | CCTGTCAATGTCACGTTCTGC | GCCACAATCCCACTGACTGA |
| *NDUFA13* | TCGAGGACTGTCGGGCTAC | TCTCCCGAAGCATCTGCAAG |
| *SDHB* | ACAAGGCTGGAGACAAACCT | GAAGTTGCTCAAATCGGGAACA |
| *SDHD* | TAGGAGGCCGAGCTCTGTT | GTGGCTCGGTGACAAGTGTA |
| *UQCRB* | TGGTAAGCAGGCCGTTTCAG | CCAGTGCCCTCTTAATGCGA |
| *UQCRC2* | ATTAGCCAAGGCATTCCGGT | GCTGGTAAGCAGGCCGATATT |
| *COX10* | TTTGTCAGTGCCGATTTTAGG | GAACAGCACAACAAGTGGCA |
| *COX6B1* | CGGGGTGCCTTTAGGATTCA | TTCTGACAGCGGTGGAAGTC |
| *ATP5A* | ACATCGTATAACTGTGGGACTGC | ATTCCTCAGCCCATGTACGC |
| *ATP5B* | CAGCAGATTTTGGCAGGTGAA | ACCCCTCACGATGAATGCTC |
| *BMP4* | GCCCGGAAGCTAGGAGC | AGCAGGATGACAGAAAACAAGG |
| *MYBPC2* | AGGGGTCGCTATCAGGTCAT | CAGCTTGTGGAACCTGCCTA |
| *MEF2C* | GCAGGTAACACAGGTGGTCT | TTCTTGCTGCCTGGTGGAAT |
| *DLL1* | ACCCTGTTCTAATGGTGCCAA | GCACACTCGCACACATAGC |
| *MYOG* | TCAGCTCCCTCAACCAGGAG | CCGTGAGCAGATGATCCCC |
| *MYF5* | CTCTGATGGCATGCCCGAAT | CTAGAAGCCCCTGGAGTTGC |

**Table S5. Antibodies for immunofluorescence**

| **Antibody** | **Source** | **Vendor** | **Cat. No.** |
| --- | --- | --- | --- |
| OCT4 | Mouse | Abcam | ab184665 |
| SOX2 | Rabbit | Abcam | ab97959 |
| NANOG | Rabbit | Abcam | ab109250 |
| SSEA4 | Mouse | Thermo fisher | 41-4000 |
| MHC (MF20) | Mouse | DSHB | AB2147781 |
| ACTN2 | Rabbit | Proteintech | 14221-1-AP |
| PAX7 | Mouse | DSHB | AB528428 |
| Nuclei (DAPI) | - | Sigma Aldrich | D9542 |
| Secondary antibody | Alexa Fluor 594 goat anti mouse | Invitrogen | A11005 |
| Secondary antibody | Alexa Fluor 488 goat anti rabbit | Invitrogen | A32731 |

**Table S6. Antibodies for western blotting**

| **Antibody** | **Source** | **Vendor** | **Cat. No.** |
| --- | --- | --- | --- |
| OXA1L | Rabbit | Proteintech | 21055-1-AP |
| Tubulin | Rabbit | Proteintech | 11224-1-AP |
| β-actin | Rabbit | Proteintech | 20536-1-AP |
| NDUFB8 | Rabbit | Proteintech | 14794-1-AP |
| SDHA | Rabbit | Proteintech | 14865-1-AP |
| UQCRC2 | Rabbit | Proteintech | 14742-1-AP |
| COX2 | Rabbit | Proteintech | 55070-1-AP |
| ATP5A1 | Rabbit | Proteintech | 14676-1-AP |
| ND1 | Rabbit | Proteintech | 19703-1-AP |
| ND2 | Rabbit | Proteintech | 19704-1-AP |
| CYTB | Rabbit | Proteintech | 55090-1-AP |
| COX1 | Mouse | Abcam | Ab14705 |
| ATP8 | Rabbit | Proteintech | 26723-1-AP |
| TOM70 | Rabbit | Proteintech | 14528-1-AP |
| NF-κB p65 | Rabbit | Proteintech | 10745-1-AP |
| Phospho-NF-κB p65 (Ser536) | Rabbit | CST | 3033 |
| Secondary antibody | HRP-Goat Anti-Rabbit IgG(H+L) | Proteintech | SA00001-2 |
| Secondary antibody | HRP-Goat Anti-Mouse IgG(H+L) | Proteintech | SA00001-1 |

**Table S7. Targeted gRNA sequences for the construction of cKO mouse**

| **gRNA** | **sequence** |
| --- | --- |
| gRNA-A1 | TACATCCCAAGACATGGCTCAGG |
| gRNA-A2 | AAAAAGCCCATGCACGCTACAGG |
| gRNA-B1 | ACATCCCAAGACATGGCTCAGGG |
| gRNA-B2 | TACTGGGCCTGTAGCGTGCATGG |
